# VAMP8-mediated MUC2 mucin exocytosis from colonic goblet cells maintains innate intestinal homeostasis

**DOI:** 10.1038/s41467-019-11811-8

**Published:** 2019-09-20

**Authors:** Steve Cornick, Manish Kumar, France Moreau, Herbert Gaisano, Kris Chadee

**Affiliations:** 1 Department of Microbiology, Immunology and Infectious Diseases, Snyder Institute for Chronic Diseases, University of Calgary, Calgary, AB Canada; 2 Departments of Medicine and Physiology, University of Toronto, Toronto, ON Canada

**Keywords:** Immunology, Diseases, Gastroenterology, Pathogenesis

## Abstract

The mucus layer is the first line of innate host defense in the gut that protects the epithelium by spatially separating commensal bacteria. MUC2 mucin is produced and stored by goblet cells that is constitutively exocytosed or hyper secreted upon sensing a threat. How coordinated mucus exocytosis maintains homeostasis in the intestinal epithelium and modulates the immunological landscape remains elusive. Here we describe how the vesicle SNARE protein VAMP8 coordinates mucin exocytosis from goblet cells. *Vamp8*^*−/−*^ exhibit a mild pro-inflammatory state basally due to an altered mucus layer and increased encounters with microbial antigens. Microbial diversity shifts to a detrimental microbiota with an increase abundance of pathogenic and mucolytic bacteria. To alleviate the heavy microbial burden and inflammatory state basally, *Vamp8*^*−/−*^ skews towards tolerance. Despite this, *Vamp8*^*−/−*^ is highly susceptible to both chemical and infectious colitis demonstrating the fragility of the intestinal mucosa without proper mucus exocytosis mechanisms.

## Abstract (web summary)

VAMP8 is a secretory pathway protein implicated in mucus secretion. Here the authors describe alterations in intestinal immune state and microbiota composition, as well as increased susceptibility to experimental colitis in mice deficient for VAMP8.

## Introduction

Mucosal surfaces are routinely challenged with a myriad of insults ranging from benign to dangerous that do not always warrant an immune response if compartmentation is successful. Within the gastrointestinal mucosa, this is accomplished by spatially separating ingested food, noxious particles, and potentially pathogenic bacteria away from the single layer of epithelial cells by the mucus layer^1,2^. This biphasic dynamic mucus layer is extensively colonized by the host microbiota in the lumen, however generally retains sterility just above the epithelial cells^3^. The primary component of this mucus layer is MUC2, an extensively O-glycosylated molecule that forms polymeric sheets to attach organisms as a decoy for colonization and provides a food source for the microbiota^4,5^. Continual depletion of the mucus layer through peristaltic movement and degradation is opposed by constitutive secretion of mucin from intestinal goblet cells. In response to an invading threat or pathogen, goblet cells can also mount a robust burst of mucus secretion thwarting the intruder away from the epithelial layer^6^.

Recently, toll-like receptors (TLRs) and inflammasomes have emerged as microbial sensors within goblet cells to initiate signal transduction pathways culminating to mucin release^7,8^. Logically microbial sensing through these germline encoded receptors appears an attractive mechanism for the host to decipher how imminent a threat is to the epithelial surface, where a thinner mucus layer delivering microbial-associated molecular patterns warrants a secretory response. Perturbation of these pathogen-recognition receptors (PRRs) leads to attenuated mucus release from goblet cells allowing for microbial interaction with the mucosal surface basally and thus exacerbating animal models of disease, such as chemical or infectious colitis^7,9^.

The importance of the mucus layer in gastrointestinal physiology is best exemplified through *Muc2*^*−/−*^ mice that display increased colonization of bacteria with the epithelial surface, increased susceptibility to colitis and development of colorectal cancer^10,11^. Lack of a mucus barrier in *Muc2*^−*/−*^ also leads to increased intestinal permeability and crypt hyperplasia^12^. Thus it is not surprising that *Muc2*^*−/−*^ mice show increased colonic colonization by pathogenic and commensal bacteria^13^. This ultimately leads to increased permeability, bacterial burden and exaggerated immune responses culminating in high disease activity in *Muc2*^*−/−*^ mice.

A likely candidate is SNARE-mediated exocytosis that facilitates vesicle–plasma membrane fusion events given the abundance of mucin vesicles stored within goblet cells. In this model, R-SNAREs, predominantly VAMPs, present on vesicles complex with Q_abc_ SNARE complexes on the plasma membrane composed of SNAP and syntaxin affording membrane fusion and expulsion of vesicle content^14,15^. We have recently reported that the protozoan parasite *Entamoeba histolytica* induces the activation of the vesicle R-SNARE VAMP8 upon interaction within goblet cells and lack of *Vamp8* leads to abrogated mucin release, increased parasitic adherence and an aggravated immune response following infection^16,17^. To fully characterize how mucin is released from intestinal goblet cells and the role coordinated mucin exocytosis plays in host physiology, we utilized *Vamp8*^*−/−*^ mice and interrogated alterations in the mucosal barrier. We build upon previous work that mucin exocytosis from goblet cells is VAMP8-dependent and perturbation of the SNARE machinery leads to morphological alterations in goblet cell structure and function. This leads to alterations in the microbiota and immune landscape skewing the mucosa to a tolerogenic phenotype to compensate for a dysfunctional barrier. Lack of mucin exocytosis increases susceptibility to chemical and infectious colitis highlighting the critical importance these mechanisms play in maintaining intestinal homeostasis.

## Results

### VAMP8 controls mucin exocytosis in goblet cells

**Fig. 1:**
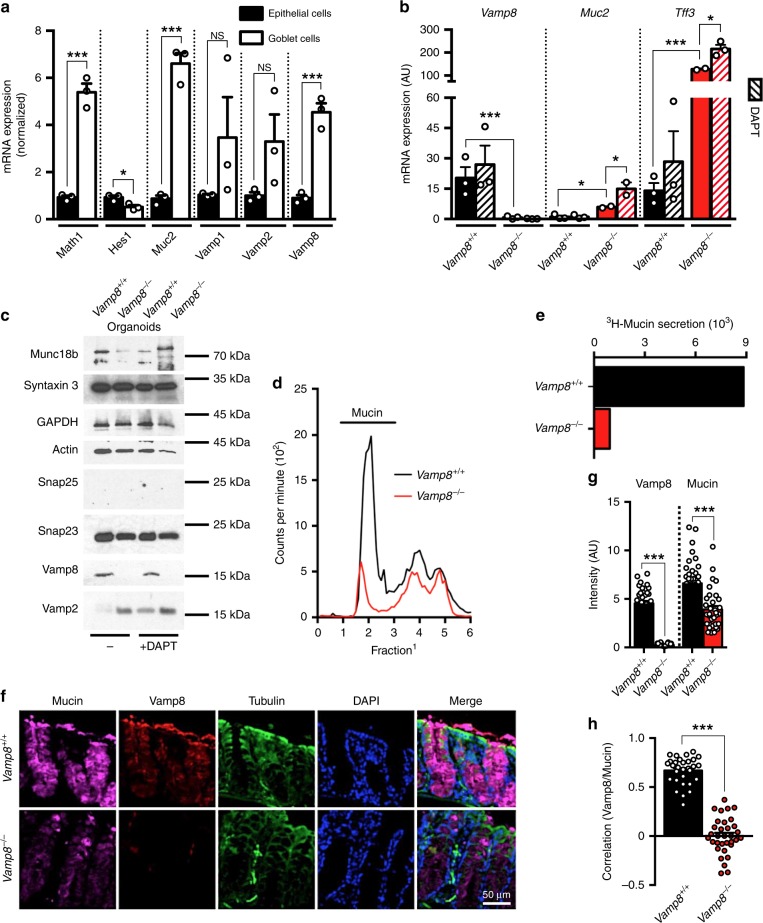
VAMP8 controls mucin exocytosis in goblet cells. **a** To assess goblet cell-specific *Vamp* isoforms, colonic epithelial cells isolated from *Atoh1*-EGFP mice were FACS sorted based on LiveCD45^−^EpCAM^+^ and sub-divided into either epithelial cells (black bars: *Atoh1-eGFP*^-^) or goblet cells (*Atoh1-eGFP*^+^) (pooled from three independent repeats with one mouse each). Relative mRNA expression of transcription factors for goblet cells (*Math1*), epithelial cells (*Hes1*), *Muc2*, and *Vamp* isoforms is shown assessed after normalization to housekeeping transcripts. **b** Colonic organoids were generated from *Vamp8*^*+/+*^ and *Vamp8*^*−/−*^ cultured for 7 days and with 5 μM DAPT for 24 h to skew organoids to a secretory lineage. (Representative data of one experiment independently repeated four times, three wells/condition.) **c** Western blot analysis was performed on colonic organoids after 7 days in culture with and without 5 μM DAPT for 24 h (three independent experiments). **d** *Vamp8*^*+/+*^ and *Vamp8*^*−/−*^ littermates (three mice per group) were metabolically labeled with ^3^H-glucosamine and mucin accumulation/secretion from pooled colonic luminal contents assayed by Sepharose 4B column chromatography, where mucin eluted in the void volume (V_o_ fractions 15–30; two independent repeats)^7^. **e** Total ^3^H-mucin secretion was inferred from assessing the area under the curve between fractions 15 and 30 from **d**. **f** Mouse colonic sections were immunostained for mucin (magenta; detecting backbone protein structure), Vamp8 (red), and Tubulin (green) in *Vamp8*^*+/+*^ and *Vamp8*^*−/−*^ littermates (three independent experiments, three mice per genotype, scale bar 50 μm). **g** The fluorescence intensity by surface area of both Vamp8 and mucin in colonic tissue sections was assessed from ROIs within the epithelium (pooled from three independent experiments, three mice per genotype, >10ROIs per mouse). **h** Colocalization of Vamp8 and Mucin within colonic tissue sections was assessed from ROIs within the epithelium and calculated for the Pearson correlation coefficient with a value of 1 being perfect colocalization, 0 being no colocalization, and −1 being exclusively localized (three mice per group, >10 ROIs per mouse). Bars indicate mean ± standard error of the mean. *P < 0.05, ***P < 0.001 Unpaired two-tailed Student's *T*-test **b**, **g**, **h**, one-way ANOVA **b**

Based on our previous reports of VAMP8 participating in mucin secretion in response to a pathogen^17^, we sought to identify the participation and expression of other Vamp isoforms in goblet cells. To directly interrogate goblet cell transcripts in the colonic epithelium, we utilized Atoh1-eGFP mice that specifically express eGFP in goblet cells (Supplementary Fig. 1A)^18^. As expected, Atoh1-eGFP goblet cells express specific markers of goblet cells, such as Muc2, −5ac, 6 as well as Tff3, and are devoid of the opposing cell fate transcription factor Hes1 (Fig. 1a). Using this technique, we identified that *Vamp8* is the predominant isoform expressed in FACS sorted mouse goblet cells. Intestinal organoids derived from *Vamp8*^*+/+*^ expressed *Vamp8*, whereas *Vamp8*^*−/−*^ did not and skewing organoids to a goblet cell phenotype had no effect on *Vamp8* expression (Fig. 1b). To confirm successful commitment to the goblet cell lineage, cultured organoids grown for 7 days then treated with DAPT for 24 h showed an increased mRNA expression of the goblet cell markers *Muc2* and *Tff3* (Fig. 1b). Interestingly, *Vamp8*^−*/−*^ expressed more *Muc2* and *Tff3* than *Vamp8*^*+/+*^ counterparts. *Vamp8*^−*/−*^ organoids displayed aberrant expression of Vamp2 with normal expression of other SNAREs Snap23, Syntaxin 3, and Munc18b with DAPT having no effect on SNARE expression (Fig. 1c).

By metabolically labeling Muc2 mucin in vivo with ^3^H-glucosamine and tracking basal high M_r_ V_0_ mucin secretion by Sepharose 4B column chromatography, *Vamp8*^*−/−*^ secreted 9-fold less mucin than *Vamp*^*+/+*^ littermates (Fig. 1d, e). The importance of VAMP8 in human goblet cells LS174T was realized in both PMA-induced and constitutive mucin secretion, which was severely attenuated in knockdown cells and unaffected by tetantus toxin to cleave neuronal VAMP isoforms (Supplementary Fig. 1B, C). In mouse colonic sections, Vamp8 co-localized to mucin granules, however, this was absent in *Vamp8*^*−/−*^ (Fig. 1f). *Vamp8*^*−/*−^ colonic tissues expressed less Vamp8 and mucin compared to WT littermate controls as assessed by fluorescence intensity (Fig. 1g). Vamp8 and mucin was also positively correlated in *Vamp8*^*+/+*^ colonic tissue sections with a strong colocalization coefficient whereas *Vamp8*^*−/−*^ had no correlation (Fig. 1h). VAMP8 co-localized to mucin vesicles in LS174T cells that was often encased within an actin-rich structure, likely the goblet cell theca (Supplementary Fig. 1D). In LS174T cells VAMP8 and mucin colocalized strongly within the goblet cell theca, however less so in the cell cytoplasm outside of the theca (Supplementary Fig. 1E).

### Alterations to the mucosal barrier in *Vamp8*^*−/−*^

**Fig. 2:**
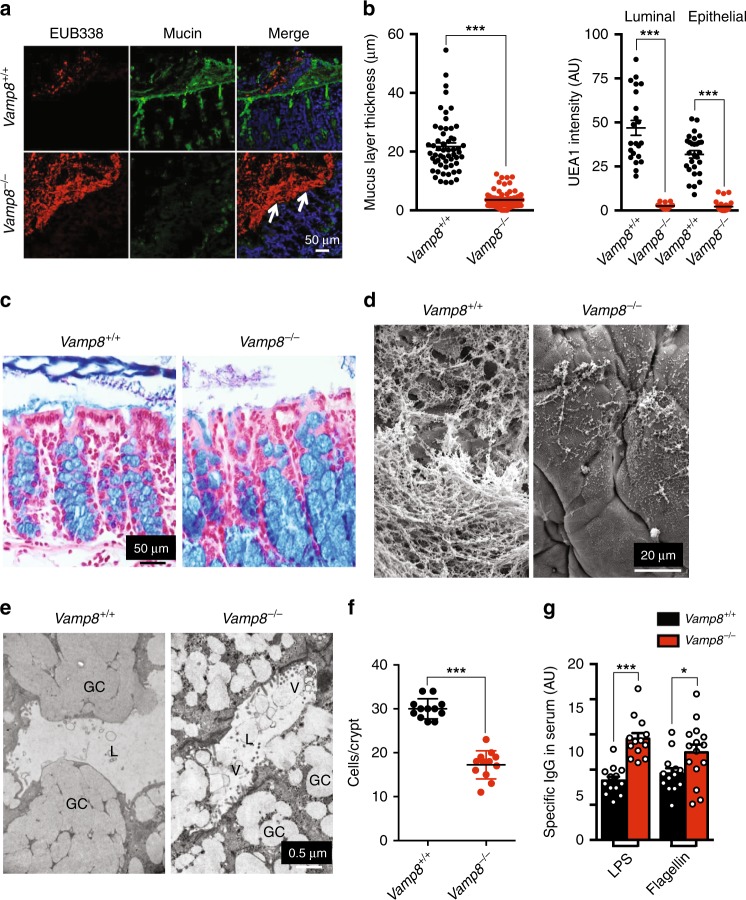
Alterations to the mucosal barrier in *Vamp8*^*−/−*^. **a** Commensal bacteria was visualized with the ubiquitous FISH probe EUB338 (red) and mucin with UEA1-Fucose lectin (green) on Carnoy’s fixed colon tissues (four independent experiments, three mice per genotype; scale bar 50 μm). Note the paucity of mucus in *Vamp8*^*−/−*^ with bacteria in contact with the surface epithelium (arrows). **b** The thickness of the mucus layer adjacent to the apical epithelial surface was measured on multiple ROIs as defined from the distance from the apical surface to nearest detectable EUB338+ bacteria (pooled from four independent experiments, three mice per genotype, >4 ROIs per mouse). The intensity of UEA1 fucose in both the lumen and epithelial cell layer was assessed (six mice per genotype, >4 ROIs per mouse). **c** Colon sections were stained with Alcian blue to visualize secreted and mucin in goblet cells (three independent experiments, three mice per genotype, scale bar 50 μm). **d** Scanning electron microscopy of the epithelial surface was performed on unwashed colon segments showing abundant mucin strands in *Vamp8*^*+/+*^ but not in *Vamp8*^*−/−*^ littermates (four independent experiments, two mice per genotype, scale bar 20 μm). **e** Transmission electron microscopy was used to observe ultrastructure anomalies in goblet cells (GC) and mucin vesicles (V) in the lumen (L) of *Vamp8*^*−/−*^ (four independent experiments, two mice per genotype, scale bar 0.5 μm). **f** The number of epithelial cells per crypt was quantified by counting histological specimens manually (three mice per genotype, >3 crypts per section). **g** Circulating IgG antibodies against LPS and Flagellin were quantified in the serum from *Vamp8*^*+/+*^ and *Vamp8*^*−/−*^ littermates by ELISA (pooled from three independent experiments, five mice per genotype). Bars indicate mean ± standard error of the mean. *P < 0.05, ***P < 0.001 Unpaired two-tailed Student's *T*-test **b**, **f**, **g**

Given the consequences on mucin secretion observed in *Vamp8*^*−/−*^ mice, we interrogated if these animals have a functional mucus layer. *Vamp8*^*+/+*^ littermate colons showed an organized mucus layer and restricted commensal bacteria to the outer mucus layer, while the inner mucus layer remained sterile (Fig. 2a). In contrast, *Vamp8*^*−/−*^ littermates lacked an inner mucus layer that was significantly thinner with bacteria often seen in direct contact with epithelial cells (Fig. 2a, b). We also observed changes in mucin glycosylation where fucose lectin only weakly stained colonic mucins in both the lumen and epithelial cell layer in *Vamp8*^*−/−*^ compared to WT littermate controls (Fig. 2a, b; green channel). To confirm this, we isolated colonic epithelial cells and stained CD45^−^EpCAM^+^MUC2^+^ goblet cells with either WGA to detect GalNAc/Galactose or UEA1 to detect fucose (Supplementary Fig. 2A). *Vamp8*^*−/−*^ mice had a decrease in fucose^+^ mucins with a compensatory increase in GalNAc/Galactose. Colonic organoids derived from *Vamp8*^*−/−*^ displayed a significant decrease in *Fut2* expression compared to *Vamp8*^*+/+*^, as well as a trend towards less C3gnt6 expression (Supplementary Fig. 2B, C). By histological staining, *Vamp8*^*−/−*^ goblet cells appeared to take on a bloated phenotype with granule–granule coalescence likely due to aborted apical exocytosis (Fig. 2c). Strikingly, the mucosal surface of *Vamp8*^*−/−*^ appeared bare, with exposed microvilli and disrupted apical surfaces in contrast to the rich mucus layer present in WT littermates (Fig. 2c). Closer examination by scanning and transmission electron microscopy showed drastic alterations in mucin strands present on the surface mucosa (Fig. 2d) and goblet cell integrity and organization, where entire mucin granules were observed in the lumen indicative of non-classical secretion mechanisms (Fig. 2e). Ultrastructural quantification of mucin granule size revealed *Vamp8*^*−/−*^ goblet cells contain smaller granules than littermate controls suggesting Vamp8 is possibly utilized in granule–granule fusion (Supplementary Fig. 2D). The total number of epithelial cells within the colonic crypt in *Vamp8*^*−/−*^ was also reduced compared to WT littermates (Fig. 2f). Interestingly, FITC-dextran permeability was not significantly different in *Vamp8*^*−/−*^ littermates despite drastic changes to the mucosal layer (Supplementary Fig. 3A). However, transepithelial resistance of colonic explants in Ussing chambers showed a rapid loss in TER and lack of response to forskolin (Supplementary Fig. 3B, C). Integrity of tissue and lack of a mucin layer ultimately led to increased microbial sensing of commensal bacteria resulting in increased IgG antibodies against LPS and Flagellin in the serum of *Vamp8*^−*/−*^ (Fig. 2g).

### Improper mucin secretion in *Vamp8*^*−/−*^ is pro-inflammatory

**Fig. 3:**
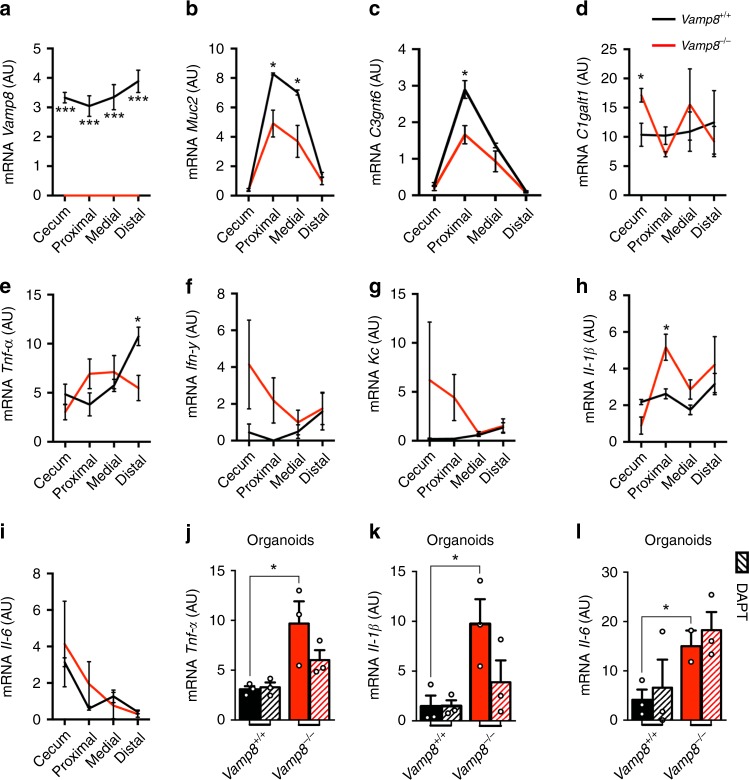
*Vamp8*^*−/−*^ exhibits a mild pro-inflammatory phenotype due to improper mucin secretion. **a** In vivo*, Vamp8* mRNA expression assessed in *Vamp8*^*+/+*^ and *Vamp8*^*−/−*^ littermates by qPCR on cecum, proximal, medial, and distal colon segments (representative data of one experiment independently repeated three times, >3 mice per group.) Goblet cell genes *Muc2* **b** *C3gnt6* **c** and *C1galt1* **d** were analyzed by qPCR. The pro-inflammatory genes *Tnf-α* **e**, *Ifn-γ* **f**, *Kc* **g**, *Il-1β* **h**, and *Il-6* **i** were measured by qPCR. **j**–**l** Colonic organoids derived from *Vamp8*^*+/+*^ and *Vamp8*^*−/−*^ littermates were analyzed for mRNA expression of *Tnf-α* **j**, *Il-1β* **k**, and Il-6 **j** with (hashed bars) and without skewing to a goblet cell lineage with 5 μM DAPT for 24 h after 7 days in culture (representative data of one experiment independently repeated two times, three wells/condition). Bars indicate mean ± standard error of the mean. *P < 0.05, ***P < 0.001. Unpaired two-tailed Student's *T*-test **a**–**l**

qPCR was performed on various regions of the colon to assess if *Vamp8*^*−/−*^ had alterations in key goblet cell transcripts. Expectedly, *Vamp8*^*−/−*^ mice had no expression of *Vamp8* and expressed less *Muc2* in the proximal and medial colon (Fig. 3a, b). This was in contrast to intestinal organoids derived from *Vamp8*^*−/−*^ which displayed the opposite phenotype (Fig. 1b). Since the protective functions of mucin are conferred by extensive O-linked glycosylation, the expression of the two most abundant core structure glycosyltransferases, core 1- and core 3-, were assessed. Similar to Muc2 expression, there was a mirrored decrease in core 3-glycosyltransferase *C3gnt6* that is critical in attaching primary glycans to *Muc2* resulting in a thicker mucus layer, while the core 1- glycosyltransferase *C1galt1* was unaffected (Fig. 3c, d)^19^. The goblet cell differentiation marker *Klf4* was elevated in *Vamp8*^*−/−*^ compared to WT littermates however, other goblet cell markers including *Spdef*, *Gfi1*, and *Atoh1* were similar (Supplementary Fig. 4A–E). Colonic organoids showed similar levels of goblet cell transcription factors between *Vamp8*^*+/+*^ and *Vamp8*^*−/*−^, however upon skewing to a goblet cell lineage, *Vamp8*^−*/−*^ were more radically modulated to a goblet cell phenotype (Supplementary Fig. 4F–J). Pro-inflammatory cytokines *Tnf-α, Ifn-γ*, *Kc*, and *Il-6* were trending towards a slight increase in *Vamp8*^*−/−*^ particularly in the proximal colon as were other cytokines related to microbial sensing, such as *Il-22*, *Il-23*, and *Il-33*, however only *Il-1β* was significantly elevated (Fig. 3e–i, Supplementary Fig. 4K–M). *Vamp8*^*−/−*^ organoids had higher expression of *Tnf-α, Il-1β*, and *Il-6* compared to *Vamp8*^*+/+*^, however this was irrespective of the goblet cell phenotype (Fig. 3j–l). Differences in cytokine expression, particularly *Tnf-α* and *Il-6*, in whole thickness colonic intestinal biopsies compared to organoids likely stems from multiple cell populations being represented in whole thickness qPCR analysis, which masks epithelial cell-specific modulation. These results depict a colonic epithelium in *Vamp8*^*−/−*^ that retains normal differentiation of goblet cells yet exhibits a mild pro-inflammatory phenotype due to increase in microbial sensing.

**Fig. 4:**
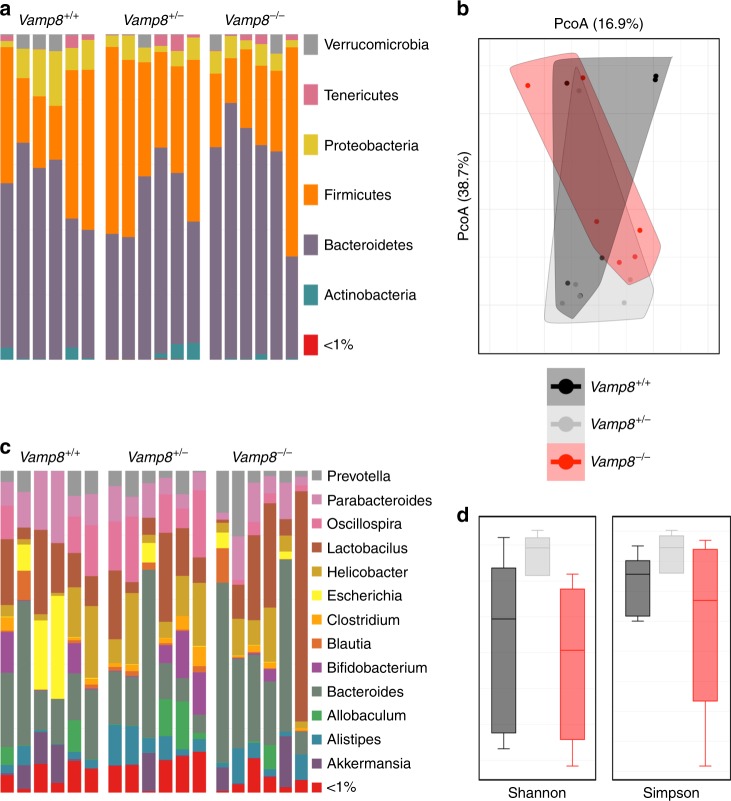
Microbiota differences in *Vamp8*^*−/−*^. 16S Sequencing was performed on fresh stool samples of *Vamp8*^*+/+*^*, Vamp8*^*+/*−^, and *Vamp8*^*−/−*^ littermates (six mice per group). **a** Phyla diversity was similar between all three groups with no significant changes between groups. **b** Beta diversity as assessed by Bray–Curtis showed a similar diversity between groups. **c** Genus abundance varied slightly between *Vamp8*^*+/+*^ and *Vamp8*^*−/−*^ littermates. **d** Alpha diversity as assessed by Shannon and Simpson was similar between *Vamp8*^*+/+*^ and *Vamp8*^*−/−*^, however *Vamp8*^*+/−*^ was more consistent

### Microbiota diversity in *Vamp8*^*+/+*^ and *Vamp8*^*−/−*^ littermates

We hypothesized that low level inflammation and altered glycosylation of mucin would impact microbial diversities in *Vamp8*^*−/−*^ and *Vamp8*^*+/+*^ littermates. 16S sequencing from stool samples showed no differences at the phyla level in the microbial communities with similar alpha and beta diversities (Fig. 4a–d). However, at the genus level, several potential pathogenic species were more abundant in *Vamp8*^*−/−*^ including *Pasteurella, Helicobacter*, and *Escherichia* (Supplementary Table 1). Additionally, *Vamp8*^*−/−*^ had greater abundance of bacteria known to modulate the thickness of the mucus layer including *Parabacteroides, Allobaculum*, and *Adlercreutzia*. There was also a decrease in beneficial bacterial species *Dehalobacterium*, *Lactobacillus*, and *Propionibacterium*. Overall, this change in commensal bacteria depicts a landscape where *Vamp8*^−*/−*^ has a mildly deleterious microbiota that favors pathogenic species, modulates mucus thickness and hinders the growth of beneficial bacteria.

### Barrier defects in *Vamp8*^*−/−*^ drives a tolerogenic phenotype

**Fig. 5:**
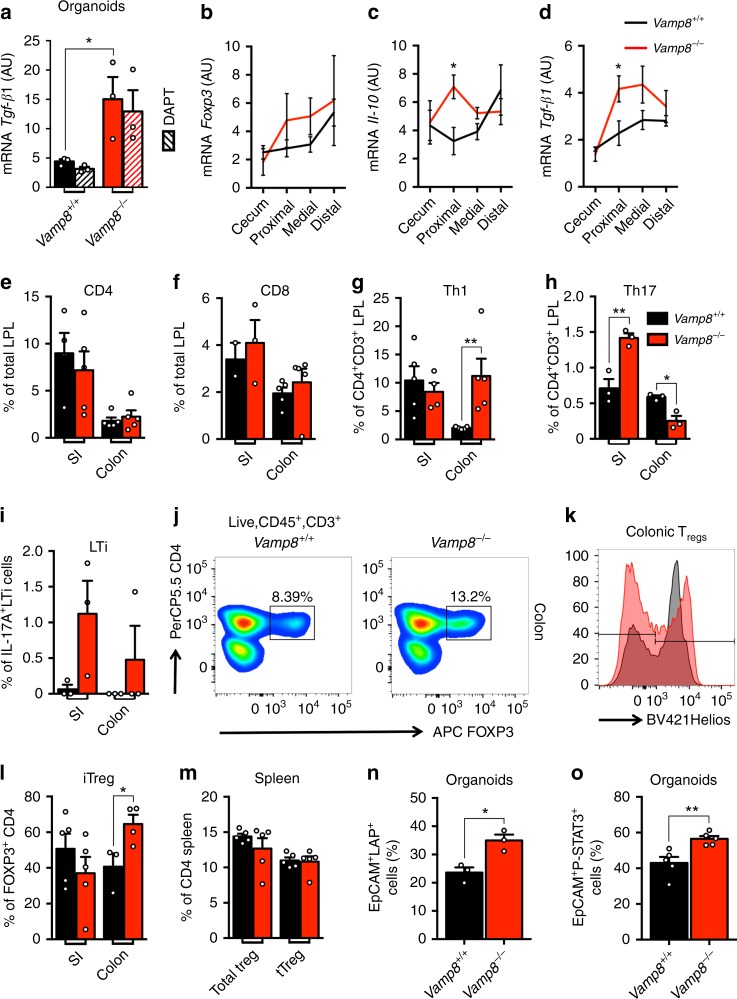
Barrier defects in *Vamp8*^*−/−*^ drives a tolerogenic phenotype. **a** Colonic organoids derived from *Vamp8*^*+/+*^ and *Vamp8*^*−/−*^ littermates were analyzed for mRNA expression of *Tgf-β1* by qPCR with (hashed bars) and without 5 μM DAPT for 24 h after 7 days in culture (representative data of one experiment independently repeated two times, three wells/condition). Tolerogenic markers *Foxp3* **b**, *Il-10* **c**, and *Tgf-β1* **d** assessed by qPCR were assayed by qPCR to determine mRNA expression (representative data of one experiment independently repeated three times with more than three mice per group). T cell profiling was performed on lamina propria lymphocytes (LPL) isolated from *Vamp8*^*+/+*^ and *Vamp8*^*−/−*^ littermates and total abundance of CD4 **e** and CD8 **f** T cells assessed (representative data of one experiment independently repeated two times with five mice per group). Abundance of Th effector CD4 T cells including Th1 (**g**; LiveCD45^+^CD3^+^CD4^+^Ifnγ^+^) and Th17 (**h**; LiveCD45^+^CD3^+^CD4^+^Il17A^+^), as well as LTi cells (**i**; LiveCD45^+^CD3^−^CD4^+^Il17A^+^) were determined in the small intestine and colonic LPL after activation in vitro with PMA/ Ionomycin with GolgiStop and GolgiPlug for 6 h (representative data of one experiment independently repeated two times with more than three mice per group). **j** Representative FACS plots of Tregs from colonic LPL. **k** Colonic Tregs (LiveCD45^+^CD3^−^CD4^+^FOXP3^+^) in *Vamp8*^*−/−*^ (red) and *Vamp8*^*+/+*^ (black) were quantified to discriminate thymic (Helios^+^) vs. periphery (Helios^−^)-derived Tregs. **l** Percentage of total Tregs devoid of the thymic marker Helios, thus termed iTregs, in small intestine and colonic LPL (representative data of one experiment independently repeated three times with more than three mice per group). **m** Spleenic regulatory T cell lineage commitment was assessed in *Vamp8*^*+/+*^ and *Vamp8*^*−/−*^ (representative data of one experiment independently repeated two times with five mice per group). Colonic organoids were cultured and analyzed by flow cytometry for expression of *Tgf-β1* (LAP; **n**) and Phospho-Stat3 **o** (representative data of one experiment independently repeated two times, three wells/condition). Bars indicate mean  ± standard error of the mean. *P < 0.05, **P < 0.01 Unpaired two-tailed Student *T*-test **a**–**i**, **l**–**o**

We sought to understand the effect of prolonged microbial sensing in the context of adaptive immunity and induction of tolerance in *Vamp8*^*−/−*^. A tolerogenic immune landscape has been shown to decrease fucosylation in the intestine and could explain the lack of fucose residues on mucin seen previously (Fig. 2a)^20^. Colonic organoids derived from *Vamp8*^−*/−*^ showed an increase in the tolerogenic cytokine *Tgf-β1* as compared to *Vamp8*^+/+^ (Fig. 5a). Likewise, in the colonic epithelium, expression of the tolerogenic cytokines *Il-10* and *Tgf-β1* were increased in *Vamp8*^*−/−*^, as well as a trend towards increased *Foxp3* (Fig. 5b–d). To assess how adaptive immune responses are modulated in the absence of mucin exocytosis, we assessed the frequency of CD4 and CD8 T cells in the small intestinal and colonic lamina propria and *Vamp8*^−*/−*^ were similar to *Vamp8*^*+/+*^ littermates (Fig. 5e, f). Given the mild pro-inflammatory state of *Vamp8*^*−/−*^, we assessed the frequency of Th1 effector T cells, where an increase was observed in the colonic LP of *Vamp8*^*−/−*^ (Fig. 5g). In addition to Th1 CD4 T cells, there was also an increase in Il-17a-producing Th17 cells in the small intestine, however, this was reversed in the colonic LP (Fig. 5h). The proportion of Il-17a producing lymphocytes was mostly CD3+, however, there was a trend towards greater Il-17a production by LTi cells (Fig. 5i). To manage these effector Th1/Th17 cells we noted a parallel increase in the abundance of FOXP3^+^-regulatory T cells in the colon that were specifically negative for the thymic marker Helios, termed iTregs (Fig. 5j, k). This appeared to be an effect of the commensal bacteria or microbial load, as these tissue-derived Tregs were not differentially expanded in the small intestinal lamina propria or in the periphery as assessed in the spleen (Fig. 5l, m). Treg expansion and production of Il-10 was region specific in the intestine as no differential expression was observed in the small intestine however, colonic CD4^+^ T cells in *Vamp8*^−*/−*^ produced significantly more Il-10 (Supplementary Fig. 5A). Epithelial cells and APC were not significantly different in expression of Il-10 (Supplementary Fig. 5B, C). In contrast, differential Tgf-β1 expression was mostly observed in epithelial and APC cells, however CD4^+^ T cells in *Vamp8*^−*/−*^ expressed similar levels (Supplementary Fig. 5D–F). Intriguingly, intestinal organoids that are sterile retained the tolerogenic phenotype producing Tgf-β1 in a STAT3-dependent manner (Fig. 5n, o). To test the importance of increased Tgf-β within the colonic epithelium in *Vamp8*^−*/−*^ on controlling Treg abundance and suppressing spontaneous inflammation in the context of increased microbial proximity, in vivo antibody neutralization with anti-TGFB (1D11) was performed. Both *Vamp8*^*+/+*^ and *Vamp8*^*−/−*^ littermates were treated for 14 days every other day with 2.5 mg/kg of either 1D11 monoclonal Tgf-β or isotype IgG control. Flow cytometry analysis on colonic LPL revealed a significant decrease in Helios^−^ iTregs in *Vamp8*^*−/−*^ mice following administration with 1D11 (Supplementary Fig. 6A). Although a trend for increase in effector Th1 and Th17 CD4^+^ cells was observed this did not reach significance (Supplementary Fig. 6B, C). Histologically there was increased epithelial apical sloughing with altered crypt architecture and inflammatory infiltrate in *Vamp8*^*+/+*^ (Supplementary Fig. 6D). In *Vamp8*^*−/−*^ mice treated with 1D11 the alteration in crypt architecture was more severe with increased inflammatory infiltrate. These results demonstrate a role for Tgf-β in controlling intestinal homeostasis through Treg abundance in the context of epithelial barrier perturbation by lack of coordinated mucin exocytosis.

### Lack of Vamp8 exacerbates DSS-induced colitis

**Fig. 6:**
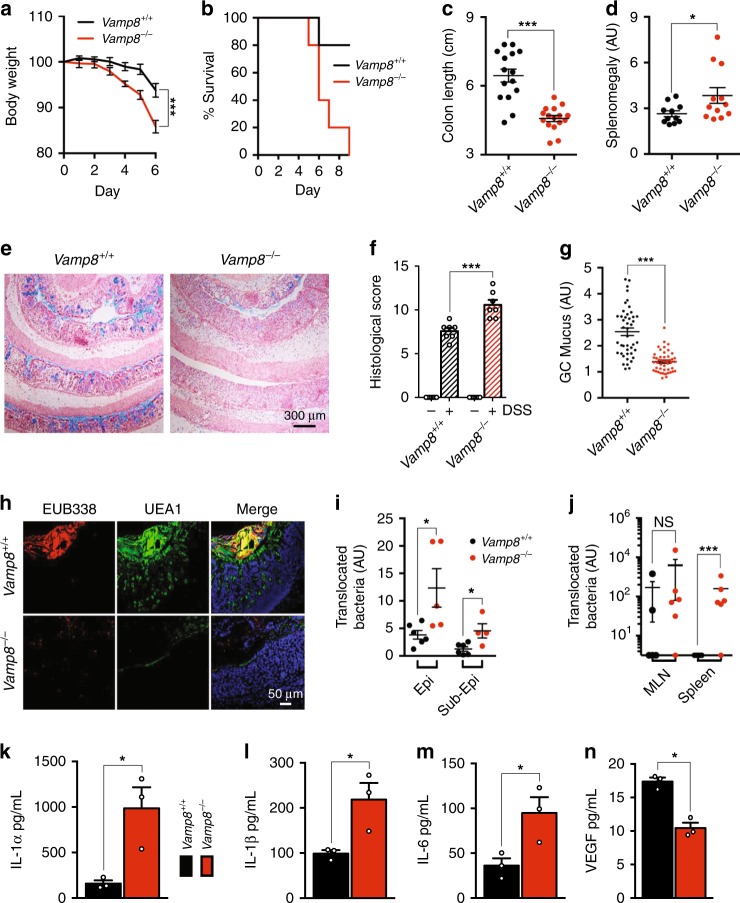
Lack of Vamp8 exacerbates DSS-induced colitis. **a** Mice were treated with 2.5% DSS in the drinking water for 5 days and monitored daily for body weight loss (pooled from three independent experiments, >3 mice per group). **b** Survival following DSS treatment was tracked and animals losing more than 20% of initial body weight were sacrificed. Classical markers of DSS colitis at day 6 were measured including shortening of the colon **c** and splenomegaly **d**. **e**, **f** Histological examination and scoring **f** by Alcian blue staining at day 6 following DSS showed hallmarks of pathology including loss of goblet cell mucin, crypt architecture, edema, and thickening of the muscularis (three independent experiments, >3 mice per group, scale bar 300 μm). **g** Goblet cell-associated mucus was quantified from alcian blue stained sections from multiple ROIs after 6 days of DSS (pooled from 9 mice per genotype, >5 ROIs analyzed per mouse by surface area). **h** Penetration and association of commensal bacteria with the epithelial cells following DSS was visualized by FISH staining with the bacterial probe EUB338 and mucin visualization with UEA1 (three independent experiments, three mice per group, scale bar 50 μm). **i** Quantification of bacteria present in the epithelial cell layer and sub-epithelial cell layer was performed from confocal FISH images (representative data from one experiment repeated three individual times with more than three mice per group). **j** To measure bacterial translocation to extra intestinal sites, spleen and mesenteric lymph nodes were homogenized and tissue lysate grown overnight with enumeration of CFU from serial dilutions counted (representative data from one experiment repeated, two individual times with more than three mice per group). Key pro-inflammatory cytokines in colonic lysate, as well was factors involved in wound healing were assessed by multiplex bead assay including Il-1α **k**, Il-1β **l**, Il-6 **m**, and VEGF **n** (representative data from one experiment repeated two individual times with three mice per group). Bars indicate mean ± standard error of the mean. *P < 0.05, ***P < 0.001. Unpaired 2-tailed Student's *T*-test **a**–**d**, **f**, **g**, **i**–**n**

Previous reports have shown Muc2 deficiency leads to increased susceptibility to DSS colitis^11^. Analogously, lack of the exocytosis machinery for mucus release in *Vamp8*^*−/−*^ littermates also led to a similar sensitivity with 3.5% DSS in drinking water for 5 days being lethal. Accordingly, we treated *Vamp8*^*−/−*^ and *Vamp8*^*+/+*^ littermates with 2.5% DSS in drinking water for 5 days and observed *Vamp8*^*−/−*^ lost 15% of their body weight after 6 days (Fig. 6a). Additionally, *Vamp8*^*−/−*^ failed to recover from DSS-induced colitis with all animals succumbing to disease by day 9 (Fig. 6b). This was in stark contrast to *Vamp8*^*+/+*^ littermates that developed a mild disease phenotype, where only 20% mortality was observed. Colitis was significantly worse in *Vamp8*^−*/−*^ as assessed by shortening of the colon and splenomegaly (Fig. 6c, d). Histopathology revealed that *Vamp8*^*−/−*^ had complete loss of crypt architecture, severe edema, thickening of the muscularis and massive neutrophil infiltrate resulting in higher pathology scores (Fig. 6e, f). There was also severe disappearance of mucin within goblet cells in *Vamp8*^*−/−*^ during DSS treatment (Fig. 6g). With the mucus layer compromised, bacteria penetrated the lamina propria and sub-mucosa with increased frequency as compared to *Vamp8*^+/+^ controls (Fig. 6h, i). This led to increased dissemination of bacteria to extra-intestinal sites including the mesenteric lymph nodes and spleen (Fig. 6j). Owing to the increased inflammation observed in *Vamp8*^−*/−*^, pro-inflammatory cytokines Il-1α, Il-1β, and Il-6 were elevated and growth factors associated with wound healing, such as Vegf were decreased (Fig. 6k–n).

### *Vamp8*^*−/−*^ is more susceptible to infectious colitis

**Fig. 7:**
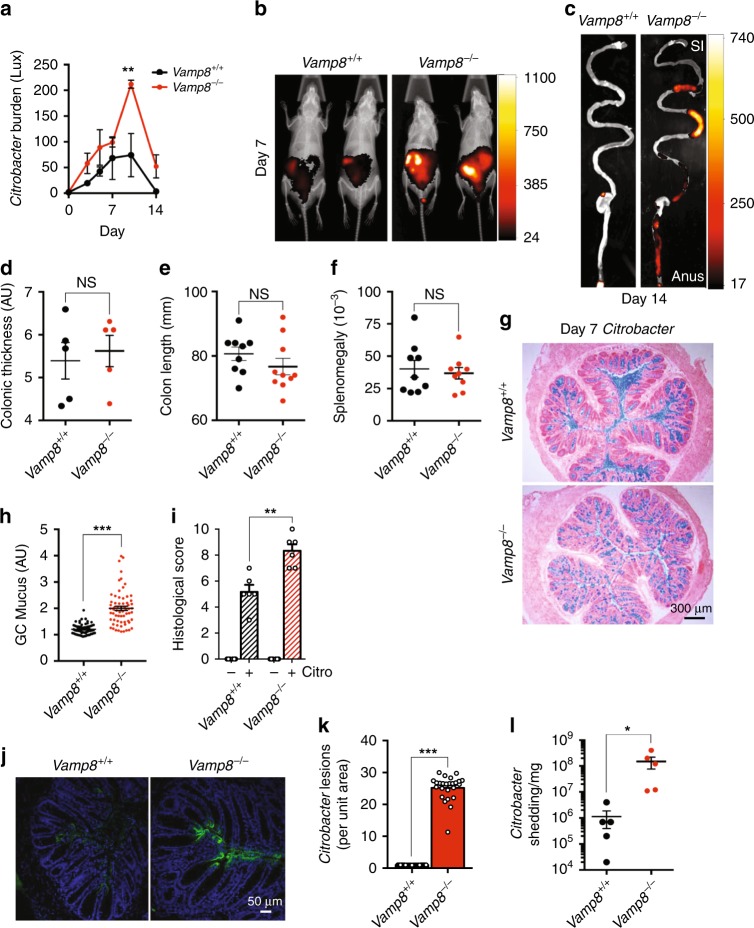
*Vamp8*^*−/−*^ littermates are more susceptible to infectious colitis. **a** Mice were infected with bioluminescent *Citrobacter rodentium* for 14 days and bacterial burden in intact animals assessed by whole body imaging (two independent experiments, four mice per group). **b** Representative images of *Vamp8*^*+/+*^ and *Vamp8*^*−/−*^ are shown at Day 7. **c** At day 14-post infection, mice were sacrificed and intestines imaged ex vivo. Gross pathology markers including colonic thickness **d**, colon length **e**, and splenomegaly **f** were measured in mice infected with *Citrobacter* at day 7 post infection (two independent experiments, five mice per group). **g** Carnoy’s fixed colon was processed for histology and stained with Alcian blue to visualize goblet cell and luminal mucin (blue) at day 7-post infection (two independent experiments, >3 mice per group, scale bar 300 μm). **h** The number of Alcian blue positive goblet cells was enumerated from multiple ROIs by surface area in *Vamp8*^*+/+*^ and *Vamp8*^*−/−*^ following *Citrobacter* infection (pooled from two independent experiments, >3 mice per group). **i** Histological scoring of *Citrobacter-*infected colonic tissue sections was performed based on mucosa thickening, epithelial cell erosion, edema, and inflammatory cell infiltrate (pooled from two independent experiments, >3 mice per group). **j** *Citrobacter* bound to epithelial cells and producing attaching and effacing lesions was visualized by confocal staining of colon tissues at day 7-post infection (two independent experiments, three mice per group, scale bar 50 μm). **k** The abundance of attaching and effacing lesions was quantified from confocal images based on ROIs by surface area (pooled from two independent experiments, three mice per group, >4 ROIs per mouse). **l** *Citrobacter* shedding into the stool was quantified by enumerating CFU at day 7-post infection (representative data from one experiment repeated two independent times, five mice per group). Bars indicate mean ± standard error of the mean. *P < 0.05, **P < 0.01, unpaired two-tailed Student's *T*-test **a**, **d**–**f**, **h**, **i**, **k**, **l**

We hypothesized that alterations to the mucus barrier through aberrant mucin exocytosis would increase susceptibility to bacterial infection. To assess this, we infected *Vamp8*^*−/−*^ and *Vamp8*^*+/+*^ littermates with *Citrobacter rodentium*, a well-established model to interrogate enterobacterial infection analogous to EPEC infection in humans. To visualize *Citrobacter* infection, mice were infected with bioluminescent *Citrobacter* and imaged in situ for up to 14 days. Bacterial load was highest at day 10 with *Vamp8*^*−/−*^ having a significant increase in *Citrobacter* burden (Fig. 7a). The localization of *Citrobacter* was similar between *Vamp8*^*−/−*^ and *Vamp8*^*+/+*^; however, total luminance was higher in *Vamp8*^*−/−*^ (Fig. 7b). Additionally, at day 14 *Vamp8*^*+/+*^ largely cleared the infection, whereas in *Vamp8*^−*/−*^ *Citrobacter* persisted localizing throughout the entire GI tract including the small intestine (Fig. 7c). Colon length, thickness, and splenomegaly were not significantly different between *Vamp8*^*−/−*^ and *Vamp8*^*+/+*^ littermates (Fig. 7d–f). Histological examination revealed massive mucus secretion in the lumen of *Vamp8*^*+/+*^ with a corresponding decrease in goblet cell resident mucin however, *Vamp8*^*−/−*^ retained mucus within goblet cells due to an absence of exocytosis (Fig. 7g, h). Histological scoring of *Citrobacter*-infected mice revealed considerably more intestinal pathology in *Vamp8*^*−/−*^ (Fig. 7i). This secretory mucin response was not observed in *Vamp8*^*−/−*^ ultimately leading to heavy colonization of *Citrobacter* with attaching and effacing lesions at the epithelial surface (Fig. 7j, k). *Citrobacter* shedding in stool samples at the peak of infection was also significantly higher in *Vamp8*^*−/−*^ as compared to *Vamp8*^+/+^ littermates (Fig. 7l).

## Discussion

The importance of mucins in health and disease has recently surged in popularity and is well recognized as a critical facet to innate immunity. Numerous studies utilizing *Muc2*^*−/−*^ mice have demonstrated the impact the intestinal mucus layer play in maintaining homeostasis in the gut and attenuating pathological responses during disease, such as *Citrobacter* infection and colitis. Lack of a functional model to describe how these mucins are released from intestinal goblet cells is therefore surprising. Valuable insight has been gained from airway goblet cells, where VAMP8 has been implicated in mucus secretion despite MUC5AC being the favored mucin component^21^. These studies have been largely motivated by understanding physiological mechanisms governing mucus release with a specific focus on airways disease with altered mucus hyper secretion such as cystic fibrosis. While the importance of VAMP8 in mucosal defense in the lung has not yet been described, mucus exocytosis in the intestine is of paramount importance given the abundant threats the gastrointestinal tract faces and heavy microbial burden. In this study, we describe that mucin exocytosis within the gastrointestinal tract solely utilizes the R-SNARE VAMP8 and elucidated how regulated mucin exocytosis is critical in homeostasis and disease.

Two mechanisms of mucin release have been proposed where goblet cells must constitutively release mucin to maintain the mucus layer and also respond to specific stimuli to execute inducible exocytosis. Our findings demonstrate that the terminal pathways culminating in mucin secretion in goblet cells is VAMP8-mediated SNARE exocytosis. By silencing VAMP8 in a human goblet cell line, we found constitutive release of mucin was severely impaired and other VAMP isoforms played a minimal role. This held true in *Vamp8*^−*/−*^ mice that secreted significantly less mucin basally than *Vamp8*^*+/+*^ littermates. Recently a role for PRRs, such as TLRs and inflammasomes has been proposed to play a role in facilitating mucin secretion in goblet cells^7,8^. Specifically, TLR1-5 respond to microbial antigens in the lumen upon deterioration of the mucus barrier and activates the NLRP6 inflammasome to induce secretion of mucin^7,8^. We propose these mechanisms function upstream of classical SNARE-mediated exocytosis. These signal cascades appear to modulate intracellular calcium levels that would potentially implicate several SNARE chaperones such as synaptotagmin that sense calcium to drive SNARE complex formation^14^. Indeed, a role for synaptotagmin-2 has been described in airway goblet cells, where intracellular calcium from the endoplasmic reticulum drives exocytosis^22^. Additionally, several members of this family localize to mucin granules and can be frequently observed at the apical surface, particularly FAM62B^23^. This therefore strongly supports the notion that exocytosis of mucin in goblet cells is SNARE-dependent. As a consequence of lack of VAMP8 in goblet cells, the mucus layer appears non-existent and permits direct contact of commensal bacteria with epithelial cells. This would facilitate constant microbial sensing of the mucosa and ultimately culminated to the mild pro-inflammatory response we observed with elevated *Il-1β, Kc*, and *Ifn-γ*. Additionally, cytokines related to microbial sensing including Il-33 and Il-22 were elevated in *Vamp8*^*−/*−24^. While Il-33 is produced by epithelial cells upon sensing bacteria, Il-22 functions downstream as an effector cytokine produced by type 3 innate lymphoid cells (ILC3) critical in dealing with pathogens and microbes^25–28^.

Given the susceptibility of goblet cells to ER stress due to MUC2 production, it would appear that improper mucin release has a grave outcome^18,29^. This can be best exemplified by our observation that goblet cells in *Vamp8*^*−/−*^ take on a pathological morphology and contain abnormal mucin granules. Further, ultrastructure anomalies were detected in *Vamp8*^*−/−*^ including the presence of intact mucin vesicles in the lumen and lack of apical epithelial cell integrity. In support of this, ex vivo intestinal explants from *Vamp8*^*−/−*^ monitored for TER in Ussing chambers deteriorated much faster than WT controls. Clearly proper secretion of mucin is required for the protective properties in mucin, which would explain why although mucus was occasionally observed in the lumen of *Vamp8*^−*/−*^ this does not result in a bona fide mucus barrier. Even though the mechanisms governing this alternative secretion are not addressed here, we propose granule extrusion as a possible cause where bloated goblet cells simply burst their content, including intact granules, into the lumen^30^. Recent work has described a specific subset of goblet cells termed sentinel goblet cells as sacrificing themselves upon reaching the top of the crypt^8^. It would be intriguing to investigate if animal models that are deficient for mucus secretion, such as VAMP8 commit greater numbers of goblet cells to this phenotype given this event appears to be TLR ligand dependent. One marked discrepancy between *Vamp8*^*−/−*^ intestinal organoids and in vivo results was mRNA expression of *Muc2* that was downregulated in vivo and upregulated in organoids. This difference likely arises from the structure of organoids with a huge accumulation of dead/dying cells within the luminal compartment. Analogous in vivo to the apical surface of the crypt, goblet cells change their transcriptomic profile to produce high levels of mucin and these GC are functionally different from crypt GCs^31^. Due to the high turnover of dead and dying cells here, we believe that organoids upregulate mucin in organoids through some similar pathways by sensing the abundant dead cells within the luminal cavity. It would be interesting to assess if GC organoids mimic the sentinel goblet cell phenotype merely based on the culture conditions and structure of the 3D organoids.

As commensal bacteria rely on mucus as food source, it was surprising that drastic differences in microbial communities in *Vamp8*^*−/−*^ was not observed^32^. Instead, subtle differences at the genus level were observed including the expansion of potential pathogens including *Pasteurella, Helicobacter*, and *Escherichia*. This appears to be consistent with several mouse models that contain barrier defects such as NOD2 and RIP2^33^. Interestingly, a large proportion of bacterial species that were up regulated in *Vamp8*^−*/−*^ are also targeted by IgA and termed colitogenic including *Erysipelotrichaceae*, *Ruminococcaceae*, and *Helicobacter*^34^. Additionally, several species that are more abundant in *Vamp8*^*−/−*^ are linked to a pro-inflammatory signature including *Erysipelotrichaceae* that is positively correlated to TNF-α levels and *Coriobacteriaceae* that is associated with Il-6^35,36^. It seems plausible that in addition to a lack of a mucus layer in *Vamp8*^*−/−*^ littermates the increase susceptibility to *Citrobacter* infection could in part be attributed to a deleterious microbiota favoring a niche of proteobacteria, as observed in other infection models, where perturbation of the microbiota with antibiotics facilitates infection^10^.

Perhaps the most unexpected result of *Vamp8*^−*/−*^ barrier dysfunction was a colonic tolerogenic response. Not only did we observe an increase in the abundance of Tregs in the LPL of *Vamp8*^−*/−*^, but there was also a concurrent increase in tolerogenic cytokines Tgf-β1 and Il-10. We suspect this is largely due to commensal bacteria as these effects were only observed in the colon that contains a high microbial burden and absent in the small intestine. While the majority of Il-10 was from Tregs in the LPL, epithelial cells contributed significant quantities of Tgf-β1. Interestingly, *Vamp8*^*−/−*^ intestinal organoids that were grown in sterile conditions retained Tgf-β1 production suggesting other mechanisms that are independent of microbiota are involved. While colonic organoids mimicked several hallmarks of in vivo epithelial cells, it should be noted that this model does not account for influences from the microbiota or immune and neuronal cells. Tgf-β1 can stimulate tissue resident T cells to express FOXP3 and maintain a tolerogenic niche^37–39^. In the lung epithelium, Tgf-β1 has been proposed to increase goblet cell abundance and promote mucus hypersecretion that could explain the greater goblet cell numbers in *Vamp8*^*−/−*^^40^. While Il-10 has been reported to induce mucin production and rectify protein misfolding in intestinal goblet cells, *Vamp8*^−*/−*^ are unable to release mucin regardless of the agonist used^41^. This is exemplified by the potent non-specific mucin inducer, PMA, where goblet cells were still attenuated for mucin exocytosis. Il-10 has been shown to suppress fucosylation in intestinal epithelial cells (IEC) and indeed *Vamp8*^−*/−*^ mice displayed less fucosylation of mucin^20^. Future studies warrant investigation on how tolerogenic cytokines and lack of the exocytosis machinery modulate glycosylation or biosynthesis of mucins and how this affects the protective functions of the mucus layer.

Since *Muc2*^−*/−*^ is highly susceptible to infectious and chemical colitis, logically abrogating the mucus secretion machinery would produce a similar phenotype. While we did not observe rampant inflammation basally or spontaneous colitis in *Vamp8*^−*/−*^, slight perturbation of the barrier had catastrophic effects. While *Muc2*^*−/−*^ can restitute following DSS and eventually recover, *Vamp8*^−*/−*^ succumb to disease by day 9^42^. This difference may be explained in the ability of *Muc2*^*−/−*^ mice to compensate by producing and secreting other Muc isoforms, predominately Muc6, that are not normally expressed in the colonic epithelium. In *Vamp8*^*−/−*^, the mechanisms governing mucin secretion are likely conserved between isoforms of mucin as no study has immunolocalized discreet mucin granules with differential cargo, for example Muc2^+^ versus Muc5ac^+^ granules. Taken together, our results demonstrate an absolute requirement for the R-SNARE VAMP8 in facilitating mucin secretion from intestinal goblet cells. In lieu of coordinated mucus exocytosis, *Vamp8*^*−/−*^ littermates failed to produce a functional mucus layer leading to increased microbial interaction with the epithelium. This defect led to a mild pro-inflammatory phenotype and skewed microbiota with greater abundance of unfavorable bacterial species. To circumvent barrier dysfunction, *Vamp8*^*−/−*^ elicited a bias mucosal immune responses skewing to a tolerogenic phenotype. Despite this, any perturbation in the barrier through infection or colitis exacerbated pathogenesis with catastrophic consequences for host physiology.

## Methods

### Cell culture and animal studies

Human adenocarcinoma colonic goblet cells, LS174T (ATCC-CL-188), were routinely passaged through nude mice to maintain a high mucin phenotype^43^. LS174T were cultured in EMEM supplemented with 10% FBS, 20 mM HEPES with 100 U/mL penicillin/streptomycin. LS174T were passaged with 0.25% trypsin/ EDTA (Thermo) once cells reached 90% confluence. For secretion experiments, LS174T were seeded in 24-well plates in triplicate at a density of 5 × 10^4^ and cultured until a confluent monolayer^44^. Doxycycline inducible LS174T shRNA silenced for VAMP8 were routinely sorted for RFP expression to maintain a high knockdown population^17^. VAMP8KD were cultured in 2 μg/mL doxycycline indefinitely to maintain knockdown given VAMP8 is a long-lived protein and non-induced cells harboring the lentiviral construct were used as WT controls. Media was replaced every 3 days and cells harvested with 0.25% Trypsin/EDTA (Invitrogen). For metabolic labeling of mucin, LS174T were seeded in 2-well dishes at 5 × 10^4^ cells/well in triplicate and allowed to reach 90% confluence.

Colonic intestinal organoids were derived from *Vamp8*^*+/+*^ and *Vamp8*^*−/−*^ littermates^45,46^. Colonic crypts were dissociated with gentle cell dissociation buffer (StemCell Technologies) and 100 crypts embedded in GFR− matrigel (BD) domes supplemented with L-WRN (ATCC CRL-3276)-conditioned media containing N2, B27, GlutaMax, SB202190, Nicotinamide, N-acetylcysteine, A83-01, and mEGF in advanced DMEM. Colonic organoids were fed every 2 days with passaging in TrypLE every 10 days. For all experiments, colonic organoids were grown for 7 days in fully conditioned media and then placed in L-WRN-conditioned media containing only N2, B27, Nicotinamide, and Glutamax for an additional 3 days. To skew colonic organoids to a goblet cell phenotype, 24 h prior to each experiment organoids were treated with 5 μM DAPT.

Six to eight-week-old *Vamp8*^*+/+*^ and *Vamp8*^*−/−*^ littermates mice on a SV129 background were bred in house^47^. *Vamp8*^*−/−*^ mice were generated by targeted gene deletion using a vector containing the entire Vamp8 gene with the second exon (mapping to amino acids 2–54 of Vamp8) replaced with IRES-LacZ and neomycin-resistance gene driven by the Pgk promoter. The neo gene was subsequently floxed out with Cre recombinase in positive carriers. Mice were kept in sterilized, filter-top cages and were maintained under specific-pathogen-free conditions with food and water ad libitum. For DSS colitis experiments, mice were administered 2.5% DSS (MP biomedicals; 36,000–50,000 Da) in the drinking water for 5 days and animal welfare carefully monitored. For *C. rodentium* infections, either Streptomycin-resistant *Citrobacter* or Lux:Lux bioluminescent *Citrobacter* was grown overnight in LB and 100 μL of overnight culture was administered by oral gavage to mice. Metabolic labeling of mucin in vivo was performed by injecting 20 μCi of ^3^H-glucosamine (Perkin Elmer) for 6 h^48^. Metabolically labeled mucin was then collected in the colonic luminal content by gentle scraping of the colon surface. Mucin was precipitated with 10% TCA, crude pellet suspended and adjusted to neutral pH. Preparations were resolved on a Sepharose 4B chromatography column with fractionation of 1 mL fractions and subsequent scintillation counting.

### Cytokine quantification and SDS–PAGE Western blot

Following the assay, cells were washed three times with ice-cold PBS and lysed in a buffer composed of 20 mM HEPES, 150 mM NaCl, 1 mM EDTA, 1% NP-40, 10 μM E64, and a protease inhibitor cocktail (Roche). Samples were then cleared by centrifuging at 14,000×*g* and supernatants quantified for protein content by BCA assay. Prior to analysis by SDS–PAGE, Laemmli sample buffer containing 5% BME was added to samples (20 μg/well) and boiled for 5 min. Samples were resolved on 10% polyacrylamide gels, wet transferred to 0.2 μm nitrocellulose and blocked with 5% skim milk. Primary antibodies diluted in PBS containing 0.1% Tween and 5% BSA were incubated overnight with blots at 4 °C. Following extensive washing, blots were incubated at RT for 2 h with secondary antibodies coupled to HRP and developed using ChemiLucent ECL detection (EMD Millipore) on film. Original unmodified scans of western blots are provided in the source data file. For quantification of cytokines and chemokines, intestinal biopsies were homogenized and sonicated in the lysis buffer listed above. Samples were normalized to 2 mg/mL and analyzed by multiplexing laser bead assay using a mouse 31plex array (Eve Technologies).

### Microscopy

For in vitro studies, 1 × 10^6^ cells/well LS174T were seeded on 5 cm^2^ No. 1.5 glass coverslips for 24 h and then fixed with 3.5% paraformadhyde followed by permeabilization with 0.35% triton. For in vivo studies, mice colons were fixed without flushing of luminal content with Carnoy’s fixative, embedded in paraffin and sectioned at 5 μm. For FISH staining, antigen retrieval was performed with proteinase K and Vamp8/Muc2 staining performed with citrate buffer. For FISH staining, slides were hybridized as previously described with the pan-bacteria probe EUB338 at 46 °C^10^. Slides were then blocked with 5% normal donkey serum and incubated overnight with primary antibodies in a humidified chamber at 4 °C. The following day, slides were washed with PBS containing 0.1% Tween and incubated at RT with fluorescent secondary antibodies, phalloidin, and DAPI (Life Technologies). Slides were mounted with Fluorosave reagent (Calbriochem) and visualized on an Olympus FV1000 scanning confocal inverted microscope. Confocal analysis was performed in Fiji (ImageJ). For mucus thickness, quantification of the area between the apical surface of the epithelium and closest detectable EUB338+ bacteria were reported for multiple random areas within a micrograph. Fluorescence intensity was measured from several separate images and multiple ROIs within an image as indicated within the figure legends (luminal and cellular). Correlation was calculated in Fiji using the Coloc package and Pearson correlation reported for and the surface area of multiple ROIs per section. Each dot on the graphs represent one ROI. For histopathology, tissue sections were blindly scored for goblet cell depletion, mucosa thickening, inflammatory cell infiltrate, loss of architecture, ulcers, and abcess^11^. For electron microscopy, mice colons were fixed in 2.5% glutaraldehyde in 100 mM phosphate buffer followed by 1% osmium tetroxide. Samples were then dehydrated through ethanol and either embedded in resin for TEM or critical point dried in HMDS for SEM. SEM images were acquired on a FEI XL30 at 30 kV and TEM acquired on Hitachi H-7650 at 120 kV.

### Flow cytometry

For analysis of lamina propria lymphocytes (LPL) and IEC, small intestine (Peyer’s patches dissected) or colon were washed extensively with PBS, flushed with PBS containing 1 mM DTT and shaken at 37 °C with HBSS containing 10 mM EDTA to strip epithelial cells from the mucosa. LPL were liberated in RPMI containing 125 units/mL collagenase at 37 °C for 1 h. Following washing with FACS buffer (PBS + 2 mM EDTA + 1%FCS) cell surface antigens were stained for 1 h, washed twice with FACS buffer and analyzed. For intracellular staining of cytokines and transcription factors, isolated LPL were stimulated in complete RPMI containing 20 ng/mL PMA, 100 ng/mL Ionomycin with GolgiStop and GolgiPlug (BD) for 6 h. Stimulated LPL were washed with FACS buffer, cell surface antigens stained and processed with FOXP3 fixation/permeabilization buffer kit according to the manufactures instructions. Cells were acquired on a BD Canto and data analyzed with FlowJo.

### 16S sequencing

DNA extraction of fecal samples was carried out using a protocol that enhances DNA recovery from microbial communities with modifications to increase quantitative recovery of bacteria across different taxa (enzymatic pre-treatment with mutanolysin, lysozyme, and proteinase K)^49^. Paired end reads of the V3 region of the 16S rRNA gene using bar coded Illumina sequencing with the modification that bar-codes are included in the forward primer^50^. Two hundred and fifty nucleotides paired-end sequencing were carried out on a MiSeq Illumina sequencer providing complete overlapping sequence reads of the V3. These overlaps were used for correcting poor quality base calls and increasing sequencing accuracy. 30–60,000 16s rRNA reads were generated per sample. The short-read library 16S rRNA gene sequencing pipeline sl1p was used^51^. All output data processing comparisons were based on OTU tables, map files, and phylogenies generated by sl1p v4.1 using the -p all -d all and -t all flags. All analyses were computed in R using phyloseq, ggplot2, and reshape2. Differential abundance analysis between groups was carried out using Bayesian estimation with R package BEST^52^.

### Colonic permeability assays

*Vamp8*^*+/+*^ and *Vamp8*^*−/−*^ littermates were gavaged with 150 μL of 80 mg/mL 4 kDa FITC-dextran (Sigma; FD4) in PBS 4 h prior to sacrifice. Mice were anaesthetized and blood was collected by cardiac punctures, which was added immediately to a final concentration of 3% acid–citrate dextrose (20 mM citric acid, 100 nM sodium citrate, 5 mM dextrose). Plasma was collected and fluorescence was quantified using a Wallace Victor (Perkin-Elmer Life Sciences) at excitation 485 nm, emission 530 nm for 0.1 s. For ex vivo quantification of TER, Ussing chambers were used to whole mount colonic segments of *Vamp8*^*+/+*^ and *Vamp8*^*−/−*^ littermate tissues^53^. Once mounted, the tissues were bathed with Krebs buffer (37 °C; pH 7.4). The serosal Krebs buffer contained 10 mmol/L glucose, and the mucosal Krebs buffer contained 10 mmol/L mannitol. Buffers were aerated and mixed by using a gas lift system (5% CO_2_ and 95% O2). Tissue responses were measured by clamping the potential difference (PD) to 0 mV by applying an Isc with a voltage-clamp apparatus (EVC-4000, World Precision Instruments, Sarasota, FL, USA). Isc was monitored throughout the experiment as the indicator of net active electrolyte transport across the tissue. After a 20-min equilibration period, the viability of the tissues was assessed by delivering an electrical field stimulation (100 V, pulse duration 500 μs, 25 Hz, 3 s) with a dual-impedance stimulator (Harvard Apparatus). At the end of all experiments, forskolin (1 μM) was applied to the serosa side of the tissues.

### RNA extraction and quantitative RT-PCR

Intestinal tissue was harvested in Trizol reagent (Thermo) and processed according to the manufactures instructions with the inclusion of RNA precipitation with LiCl_2_. cDNA preparation from 250 ng of RNA was performed using qScript first-strand cDNA synthesis kit (Quanta). qPCR was performed using SYBR green on a StepOnePlus Real-time PCR machine (Applied Biosystems). The list of primers used in this study is in Supplementary Table 2. All qPCR values were normalized to a housekeeping genes (Actin and Gapdh) and expressed graphically as a relative expression.

### Bacterial quantification

Bacteria translocation during DSS and *Citrobacter* infection was performed by homogenizing tissue lysates in sterile PBS and normalizing protein content between samples. Serial dilutions were then plated on MacConkey agar and CFU enumerated the following day. For *Citrobacter* quantification, MacConkey agar plates contained 100 μg/mL streptomycin. For whole body imaging of bioluminescent *Citrobacter*, animals were anaesthetized with 2% isofluorane carried in 2% O_2_, kept constant at 37 °C via air circulation and imaged on their ventral side on days 3, 5, 7, 10, and 14 using an in vivo Xtreme 4MP imaging platform (Bruker, Billerica, MA, USA). The imaging protocol contained three steps: reflectance imaging (2 s exposure time), bioluminescent imaging (10 s exposure time) and an additional Xray imaging step (10 s exposure time). Binning was kept constant at 4 × 4. Images from the in vivo Xtreme were acquired and analyzed using Bruker molecular imaging software MI SE (version 7.1.3.20550, Bruker, Billerica, MA, USA). *Citrobacter*-associated bioluminescence expression in the abdomen in *Vamp8*^*+/+*^ and *Vamp8*^*-/-*^ littermates was quantified by measuring the mean bioluminescence (after background subtraction) in a constant region of interest (ROI), which was kept constant over the time period of imaging.

### Statistics

Experiments presented are representative of at least three independent experiments. Statistical significance between two or more groups was assessed by one-way ANOVA where *p* < 0.05 was considered significant. For comparison between two groups Student’s *t*-test was used. For quantification of confocal images a minimum of eight images were used per condition. Results presented in histograms are displayed as the mean with the standard error of the mean for error bars.

### Ethics statement

The Health Sciences Animal Care Committee from the University of Calgary, have examined the animal care and treatment protocol (AC14-0219) and approved the experimental procedures proposed and certifies with the applicant that the care and treatment of animals used was in accordance with the principles outlined in the most recent policies on the “Guide to the Care and Use of Experimental Animals” by The Canadian Council on Animal Care.

### Reagents

Unless otherwise specified, all reagents were from Sigma-Aldrich. The following antibodies were used: Munc18b (Proteintech); SNAP23, STX3, VAMP2 (SYSY); VAMP8 (R&D); MUC2 (Santa Cruz); Th17/Treg staining kit, CD45, CD11B, CD11C, F4/80, CD103, IA-IE (BD); CD326, LAP, Il-10, IL-6, P-STAT3 (eBioscience); *Citrobacter* (Statens Serum Institute). EUB338 was from Eqixon. *Salmonella Typhimurium* flagellin was from Invivogen. Tgf-β neutralizing antibody was from BioXcell (Clone: 1D11).

## Supplementary information

Supplementary Information

Source Data

## Data Availability

The datasets generated and/or analyzed during the current study are available from the corresponding author on request. 16S sequencing data is deposited on the SRA of NCBI: Bioproject PRJNA551025.
