# Supplementary material for: VAMP8-mediated MUC2 mucin exocytosis from colonic goblet cells maintains innate intestinal homeostasis
Source: Nat Commun. 2019 Sep 20;10:4306. doi: 10.1038/s41467-019-11811-8 (PMC6754373; doi:10.1038/s41467-019-11811-8)
Supplement: Supplementary file 1 — Supplementary Information [file 41467_2019_11811_MOESM1_ESM.pdf]

VAMP8-mediated MUC2 mucin exocytosis from colonic goblet cells maintains innate intestinal homeostasis

Cornick et al

Supplementary Figure 1

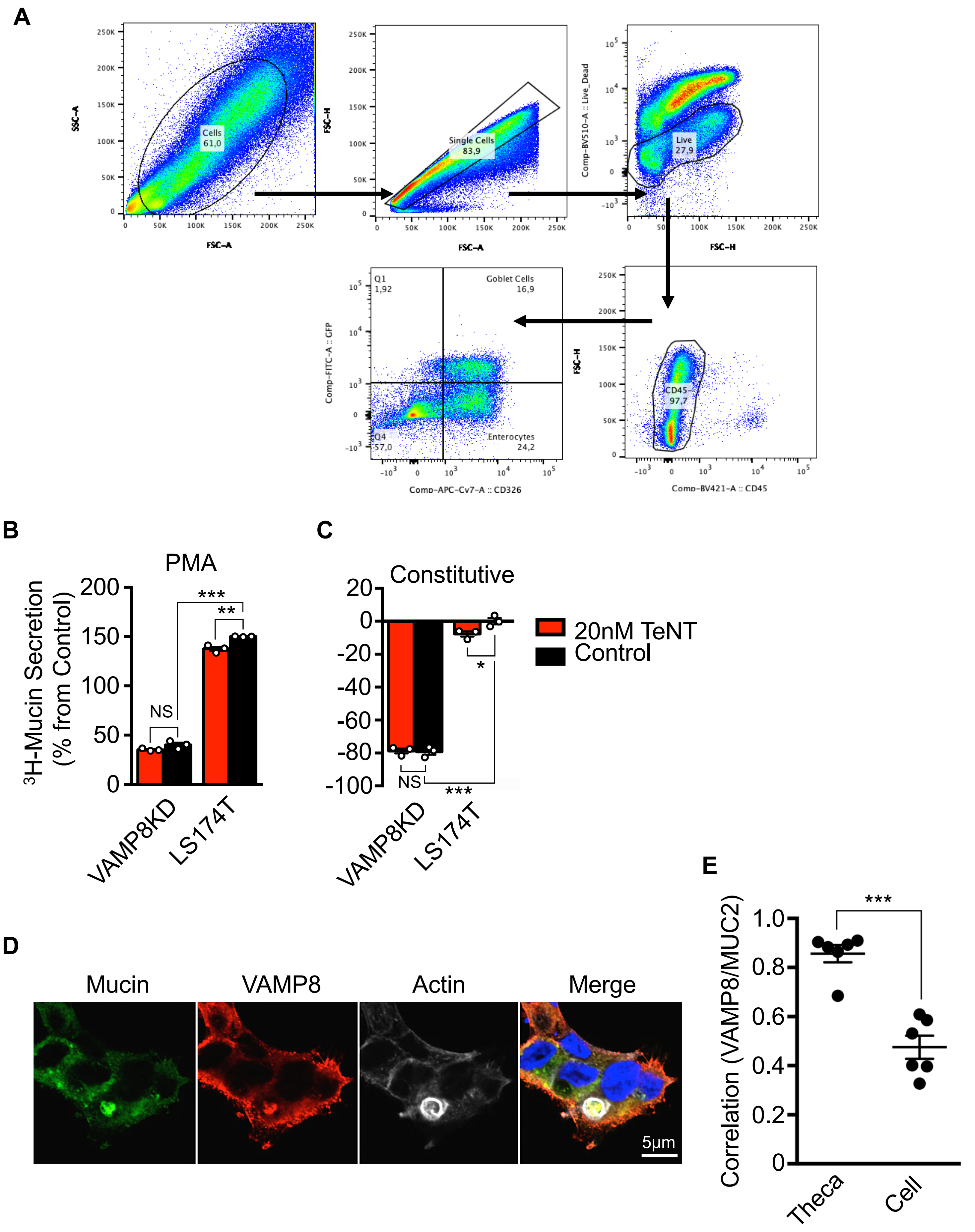

Supplementary Figure 1. *In vitro* knockdown of VAMP8 and localization. A. Colonic epithelial cells from Atoh1-eGFP mice were analyzed and sorted using this gating strategy for purification of Atoh1$^+$ goblet cells and Atoh1$^-$ enterocytes. **B & C.** LS174T cells silenced for VAMP8 expression were metabolically labeled with ^{3}H-gluosamine and mucin secretion quantified when stimulated with 5uM PMA (**B**) or basally (**C**) to assess induced and constitutive mucin secretion respectively (Mean+/-SEM; one-way ANOVA and 2-tailed Student *t*-test; 2 independent experiments, 3 wells/condition). 20nM Tetanus toxin was added to culture media 60 min prior to the secretion assay. **D.** Immunocytochemistry staining revealed VAMP8 (Red) co-localized to mucin granules (green) in LS174T cells and the granules were encased in an actin-rich (white) structure (3 independent experiments, scale bar 5μm). **E.** The correlation of VAMP8 and mucin colocalization in LS174T cells measured within an ROI containing the goblet cell theca as well as an ROI of the entire cell (Pearson's correlation; 3 independent experiments). *P<0.05, **P<0.01, ***P<0.001.

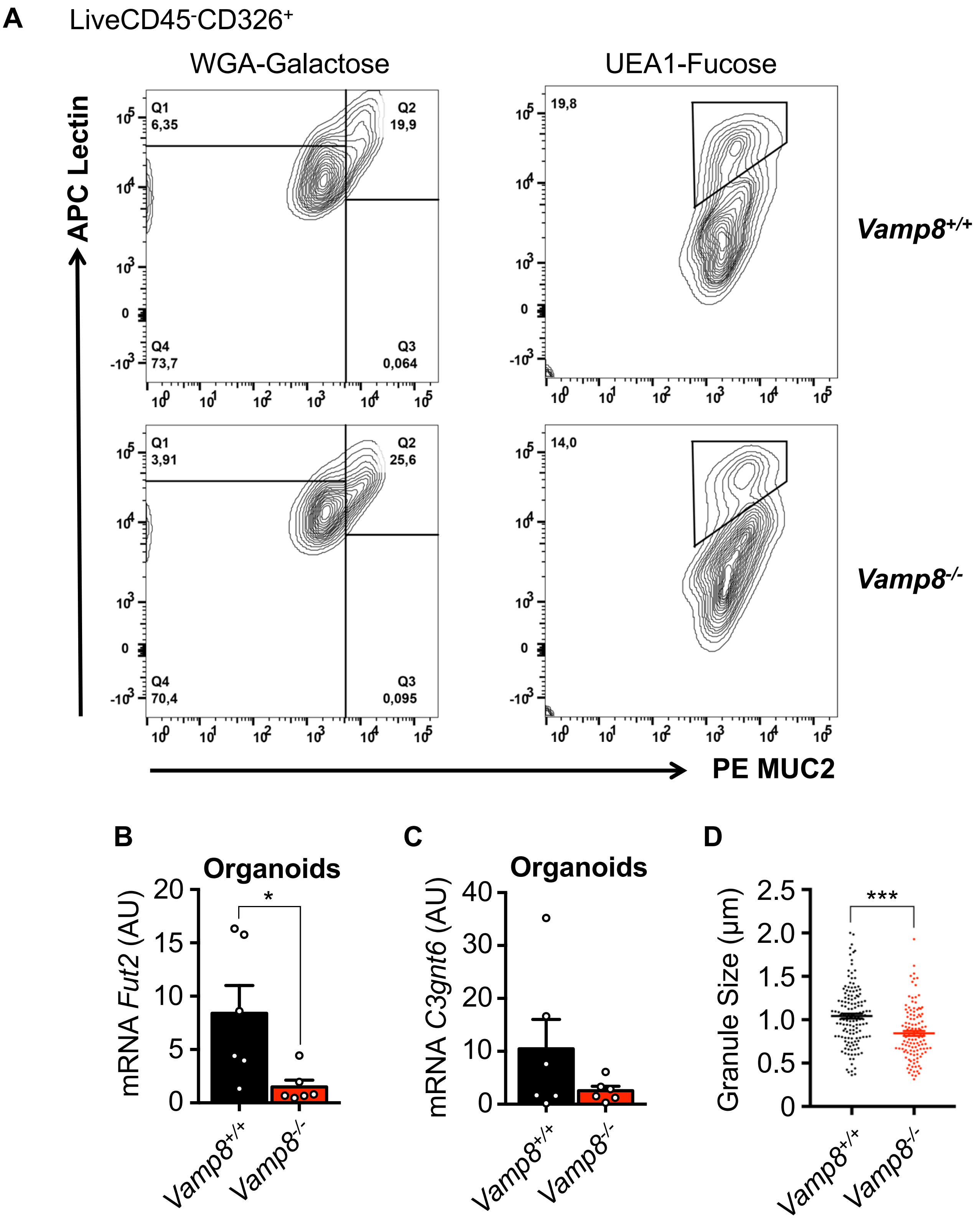

Supplementary Figure 2. Goblet cell glycan expression in *Vamp8*$^{-/-}$. A. Flow cytometry analysis was performed on isolated colonic epithelial cells (LiveCD45^{-}EpCAM^{+}) from *Vamp8*$^{+/+}$ and *Vamp8*$^{-/-}$ littermates (2 independent experiments, 3 mice per group). Abundance of either WGA (Left) or UEA1 (Right) to detect GalNAc/Galactose or fucose was quantified as well as MUC2 protein expression (X-axis). **B-C**. Colonic organoids derived from *Vamp8*$^{+/+}$ and *Vamp8*$^{-/-}$ littermates were analyzed for mRNA expression of *Fut2* (**B**) *and C3gnt6* (**C**) (Mean+/-SEM; 2-tailed Student *t*-test; 3-wells/ condition, 2 independent experiments pooled). **D.** *In vivo* mucin granules in colonic goblet cells were quantified from TEM micrographs (Mean+/-SEM; 2-tailed Student *t*-test, 4 independent experiments, >20 granules per section quantified). *P<0.05, ***P<0.001.

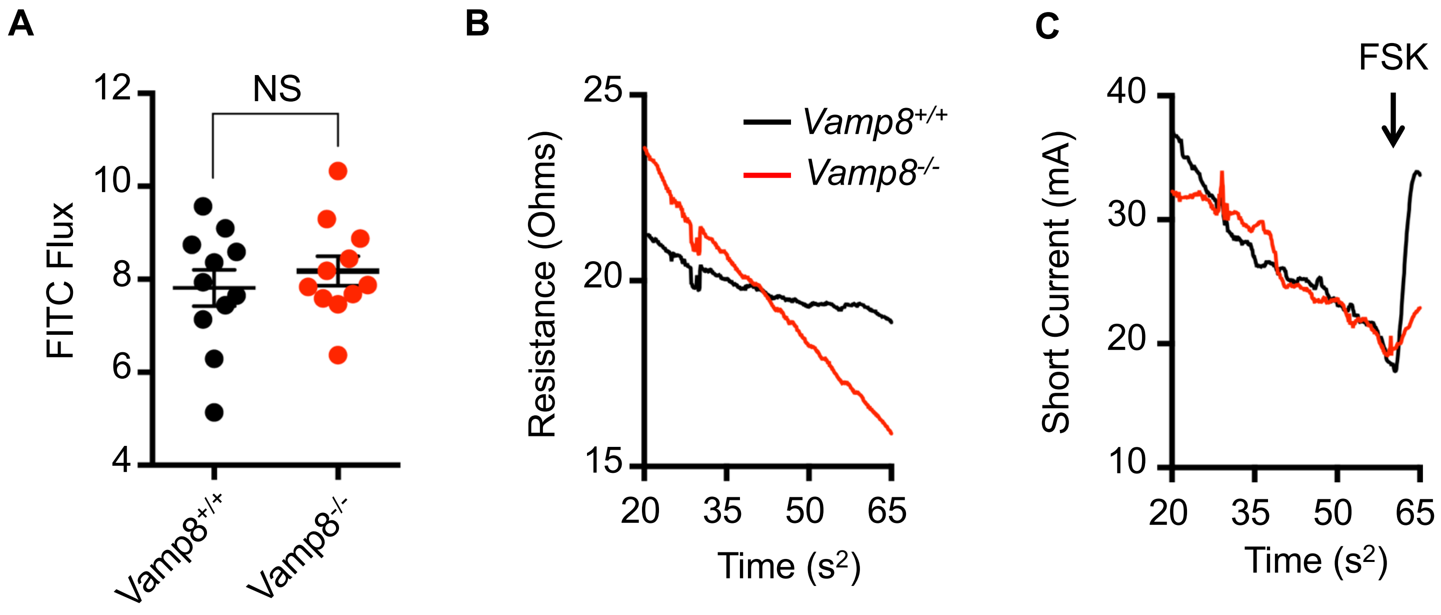

Supplementary Figure 3. Transepithelial cell resistance in *Vamp8*$^{-/-}$. A. Intestinal permeability was assessed by gavaging animals with FITC-Dextran and measuring permeability into the circulation after 3 h (Mean+/-SEM; 2-tailed Student *t*-test; 2 independent experiments pooled, >5 mice per group). Colonic explants were placed in Ussing chambers to measure resistance (**B**) and short circuit current (**C**) over 6500s (Average values from 3 mice/ condition/ chamber, 2 independent experiments). At the end of the experiment, 1uM forskolin was added to the apical side to test responsiveness of explants.

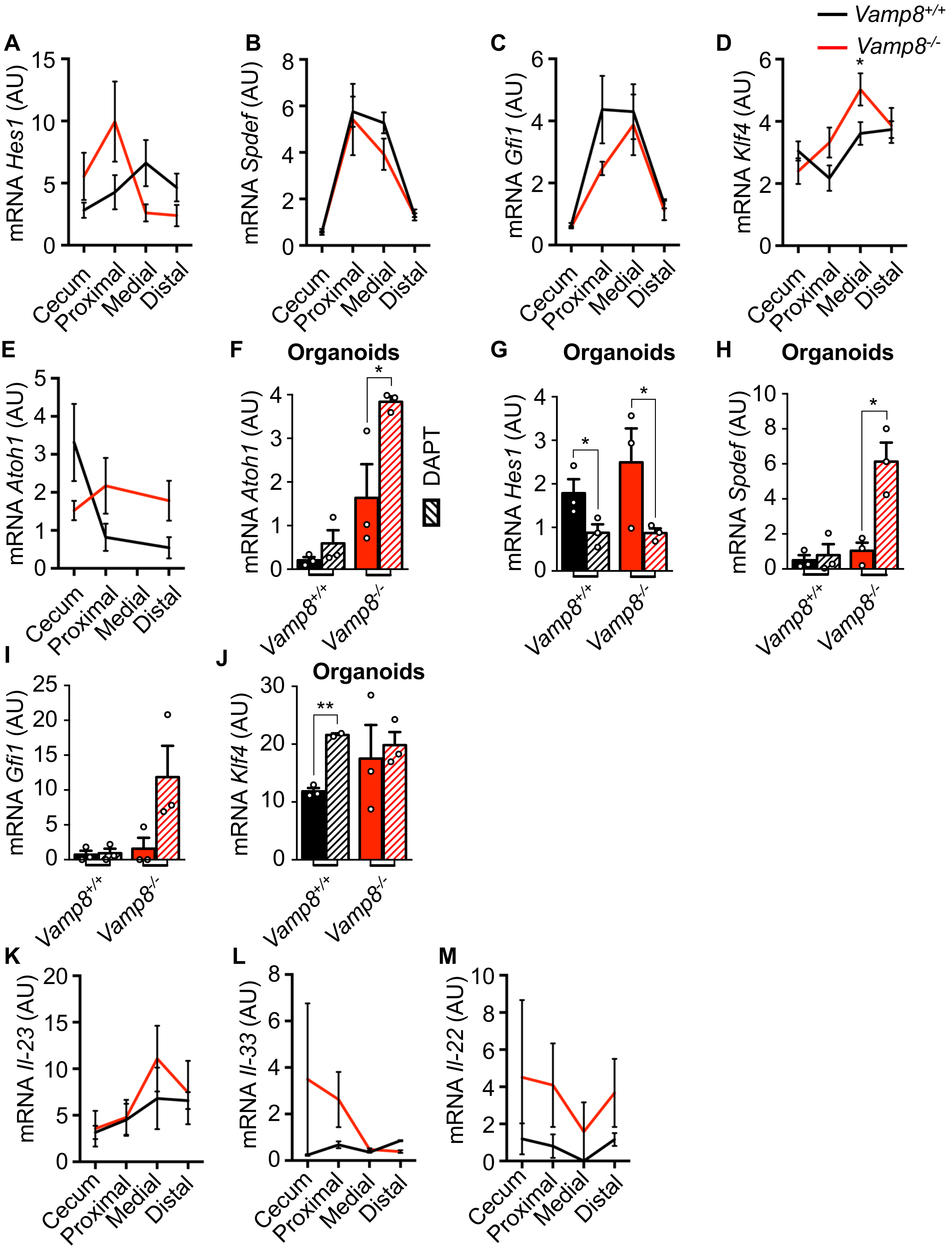

Vamp8+/+
Vamp8-/-
A
mRNA Hes1 (AU)
B
mRNA Spdef (AU)
C
mRNA Gfi1 (AU)
D
mRNA Klf4 (AU)
Cecum
Proximal
Medial
Distal
E
mRNA Atoh1 (AU)
F
Organoids
mRNA Atoh1 (AU)
DAPT
G
Organoids
mRNA Hes1 (AU)
H
Organoids
mRNA Spdef (AU)
I
mRNA Gfi1 (AU)
J
Organoids
mRNA Klf4 (AU)
K
mRNA Il-23 (AU)
L
mRNA Il-33 (AU)
M
mRNA Il-22 (AU)

Supplementary Figure 4. *In vivo* and organoid transcription factor expression. *In vivo,* colonic mRNA transcripts for genes involved in epithelial and goblet cell differentiation were assessed by qPCR including the absorptive marker *Hes1* (**A**) and secretory markers *Spdef* (**B**), *Gfi1* (**C**), *Klf4* (**D**) and *Atoh1* (**E**) (Mean+/-SEM; 2-tailed Student *t*-test, 2 independent experiments, 3 mice per group). **F-J**. Colonic organoids derived from *Vamp8*$^{+/+}$ and *Vamp8*$^{-/-}$ littermates were analyzed for mRNA expression lineage transcription factors above by qPCR with (Hashed bars) and without 5μM DAPT for 24 h after 7 days in culture (Mean+/-SEM; 2-tailed Student *t*-test; 3 wells/ condition, 2 independent experiments). **K-M.** Genes involved in microbial sensing including *Il-23* (**K**), *Il-33* (**L**) and *Il-22* (**M**) were assessed in *Vamp8*$^{+/+}$ and *Vamp8*$^{-/-}$ littermate tissues by qPCR (Mean+/-SEM; 2-tailed Student *t*-test, 2 independent experiments, 3 mice per group). *P<0.05, **P<0.01.

Phylum	Class	Order	Family	Genus	Prob.
Firmicutes	Clostridia	Clostridiales	Dehalobacteriaceae	Dehalobacterium	98.5
Firmicutes	Bacilli	Lactobacillales	Lactobacillaceae	Lactobacillus	95.27
Firmicutes	Clostridia	Clostridiales	Ruminococcaceae	N/A	99.5
Firmicutes	Clostridia	Clostridiales	Lachnospiraceae	N/A	96.74
Actinobacteria	Actinobacteria	Actinomycetales	Propionibacteriaceae	Propionibacterium	98
Actinobacteria	Actinobacteria	Coriobacteriales	Coriobacteriaceae	Adlercreutzia	98.83
Tenericutes	Erysipelotrichi	Erysipelotrichales	Erysipelotrichaceae	Allobaculum	99.2
Firmicutes	Clostridia	Clostridiales	Ruminococcaceae	Bacteroides	96.34
Proteobacteria	Gammaproteobacteria	Enterobacteriales	Enterobacteriaceae	Escherichia	95.43
Proteobacteria	Epsilonproteobacteria	Campylobacterales	Helicobacteraceae	Helicobacter	96.93
Firmicutes	Clostridia	Clostridiales	Ruminococcaceae	N/A	95.24
Firmicutes	Clostridia	Clostridiales	Lachnospiraceae	N/A	99.57
Bacteroidetes	Bacteroidia	Bacteroidales	Porphyromonadaceae	Parabacteroides	95.7
Proteobacteria	Gammaproteobacteria	Pasteurellales	Pasteurellaceae	Pasteurella	95.56

Supplementary Table 1. List of 16S bacterial genus up and downregulated in *Vamp8*$^{-/-}$

16S sequencing was performed on feces collected from *Vamp8*$^{+/+}$ and *Vamp8*$^{-/-}$ littermates. Bacterial genus that were significantly downregulated in *Vamp8*$^{-/-}$ are shown in black while genus upregulated are shown in red. Probability was calculated based on Bayesian estimation.

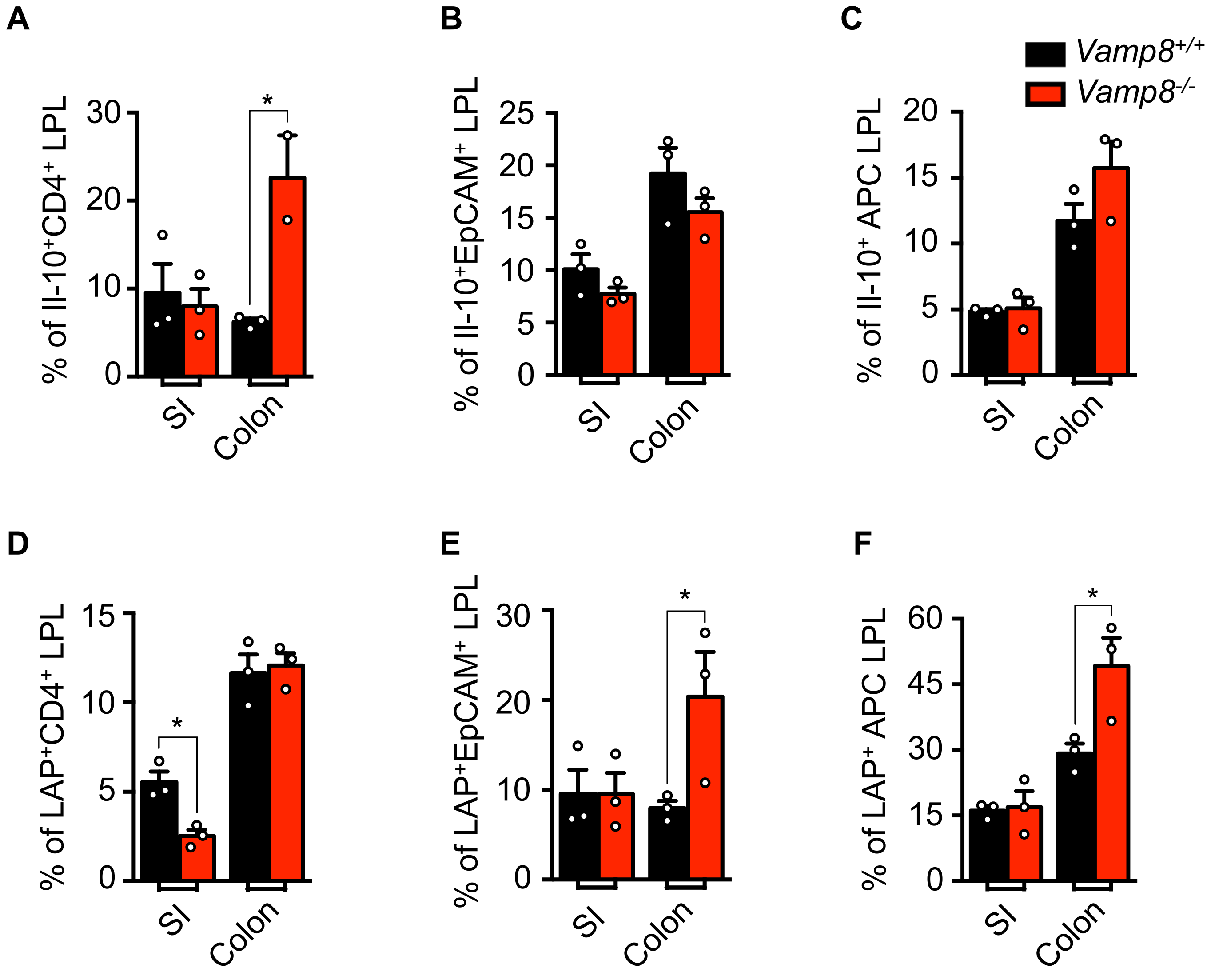

Supplementary Figure 5. IL-10 and TGFB1 expression in mucosal cells. Intracellular cytokine staining and subsequent flow cytometry analysis was performed on lamina propria lymphocytes (LPL) after *ex vivo* stimulation with PMA/ Ionomycin to evaluate Il-10 expression in $CD4^+$ T cells (**A**), $CD45^-$ $EpCAM^+$ epithelial cells (**B**) and $CD45^+MHC2^{hi}CD11c^+$ APCs (**C**). Expression of LAP (Tgf-β1) was also assessed in T cells (**D**), epithelial cells (**E**) and APCs (**F**) (Mean+/-SEM; 2-tailed Student *t*-test, 2 independent experiments, 3 mice per group). *P<0.05.

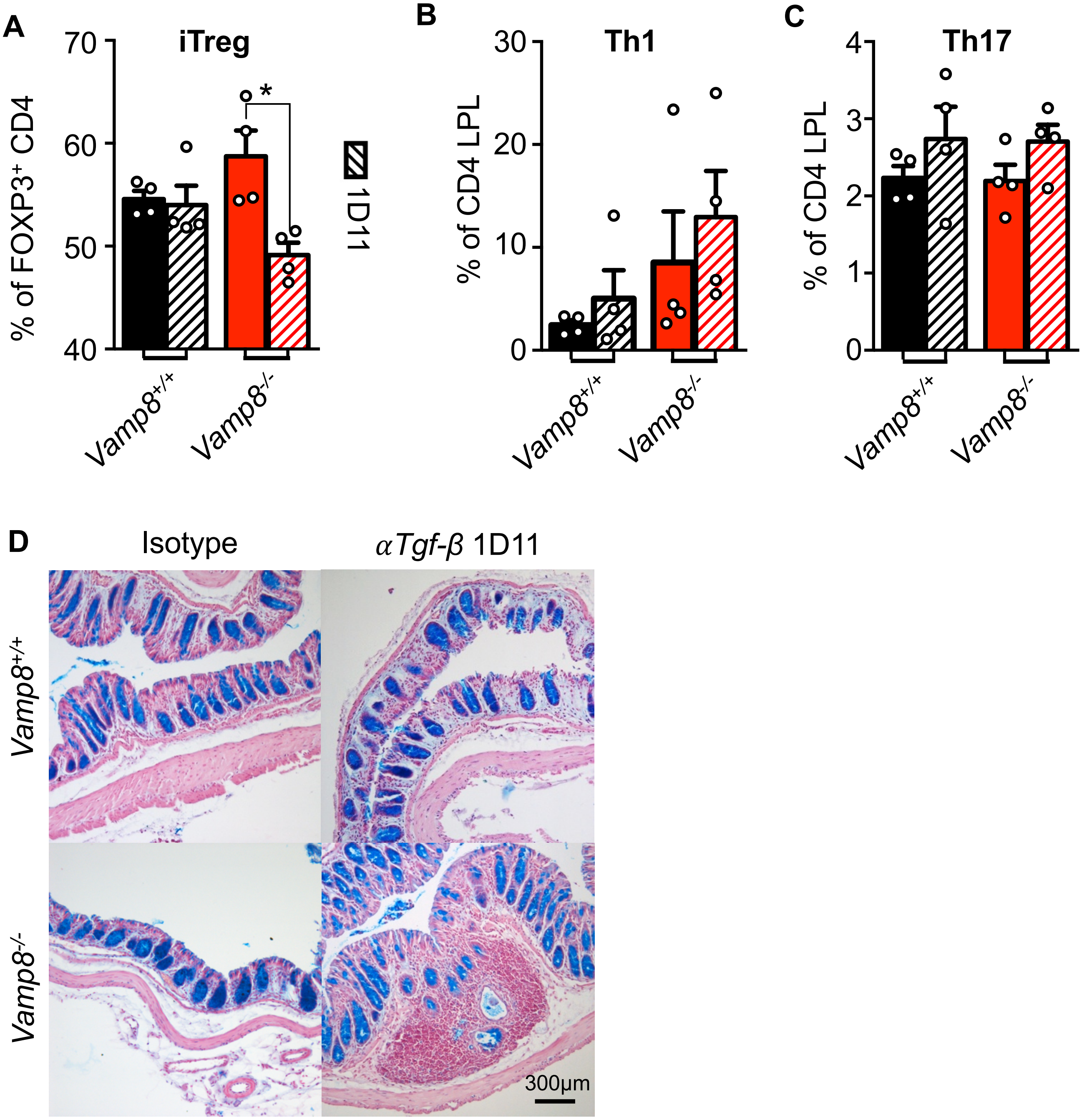

Supplementary Figure 6. TGFB controls Treg abundance and homeostasis in *Vamp8*$^{-/-}$. *Vamp8*$^{+/+}$ and *Vamp8*$^{-/-}$ littermates were IP injected with 2.5mg/kg of anti-Tgf-β neutralizing antibody (1D11; hashed bars) or isotype IgG (solid bars) every 2 days for 14 days. At day 14, colonic LPL were isolated and assessed for CD4 phenotypes including Helios^{+} iTregs (**A**), Th1 (**B**) and Th17 (**C**) by flow cytometry (Mean+/-SEM; 2-tailed Student *t*-test, 2 independent experiments, 4 mice per group). **D.** Histological examination of colonic tissues was performed by staining with Alcian blue (2 independent experiments, 4 mice per group, scale bar 300µm). *P<0.05.

Gene	Forward	Reverse
Il-10	AGTGGAGCAGGTGAAGAGTG	ACGAGGTTTTCCAAGGAGTTGT
Vamp8	GGACCACCTCCGAAACAAGA	AGGGCTCCTCTTGGCACATA
Muc2	AAACTGCTCTCTGGACTGCC	TTGGTTGGTGTGCTGAGTGT
Fut2	AGTTTCCTTGGTCCTGAACGA	CTACAGCAATCCTCTTCTGGC
B3gnt6	TTCGCGCCTTATGAGATGCT	GTGCATCGGGGTTCTGTTCT
Il-22	CCGAGGAGTCAGTGCTAAGG	CATGTAGGGCTGGAACCTGT
Il-23a	AGCGGGACATATGAATCTACT AAGAGA	GTCCTAGTAGGGAGGTGTGAAGTTG
Il-33	GCTGCGTCTGTTGACACATT	CACCTGGTCTTGCTCTTGGT
Kc	ACCTAGGCATCTTCGTCCGT	GGGCCAACAGTAGCCTTCAC
Ifn-y	GCAACAGCAAGGCGAAAAAG	ATCTCTTCCCCACCCCGAAT
Il-17a	GCCCTCAGACTACCTCAACC	CCCTGAAAGTGAAGGGGCAG
Foxp3	TGGTCTCTGCAGGTTTAGTGC	TCCAAGTCTCGTCTGAAGGC
Klf4	AGAACAGCCACCCACACTTG	CCCTGTGTGTTTGCGGTAGT
Gfi1	TTCTGAGCGCCTTGCCTTC	CGAATGTTTGGACCCTCGGAT
Spdef	CTCGCTAGAGCAGGTGCAAT	AGCCACTTCTGCACGTTACC
Il-6	TAGTCCTTCCTACCCCAATTTCC	TTGGTCCTTAGCCACTCCTTC
Muc5	CTTGTCTCAGTCCCTCCTGC	TCTGACTGTCTCCGGTGAGT
Vamp1	CGACAGTTCCGTCTGCTTCA	TGGTCATGTTGGGAGGAGGA
Vamp2	GTTTGCTTCCCTTACCCCGT	CAGTTGAGTGCCCCACATGA
Vamp7	GAACTCACGTTCCCAGGAGG	CCCTGGCAACAACAGCAAAA
Snap23	AGCGGGACAGAGTATCCGTA	CCCCTTGCTCATCCAGCATA
Gapdh	TGCCCCCATGTTTGTGATG	TGTGGTCATGAGCCCTTCC

Tff3	CTCTGTCACATCGGAGCAGTGT	TGAAGCACCAGGGCACATT
Il-1b	GCCTCGTGCTGTCGGACCCA	CTGCAGGGTGGGTGTGCCGT
Tgf-b1	TGACGTCACTGGAGTTGTACGG	GGTTCATGTCATGGATGGTGC
Atoh1	AAAGGAGGCTGGCAGCAA	TGGTTCAGCCCGTGCAT
Hes1	AAAATTCCTCCTCCCCGGTG	TTTGGTTTGTCCGGTGTCG

Supplementary Table 2. List of qPCR primers used in this study
