# Supplementary figures and images for: VAMP8-mediated MUC2 mucin exocytosis from colonic goblet cells maintains innate intestinal homeostasis
Source: Nat Commun. 2019 Sep 20;10:4306. doi: 10.1038/s41467-019-11811-8 (PMC6754373; doi:10.1038/s41467-019-11811-8)

## Slide 1
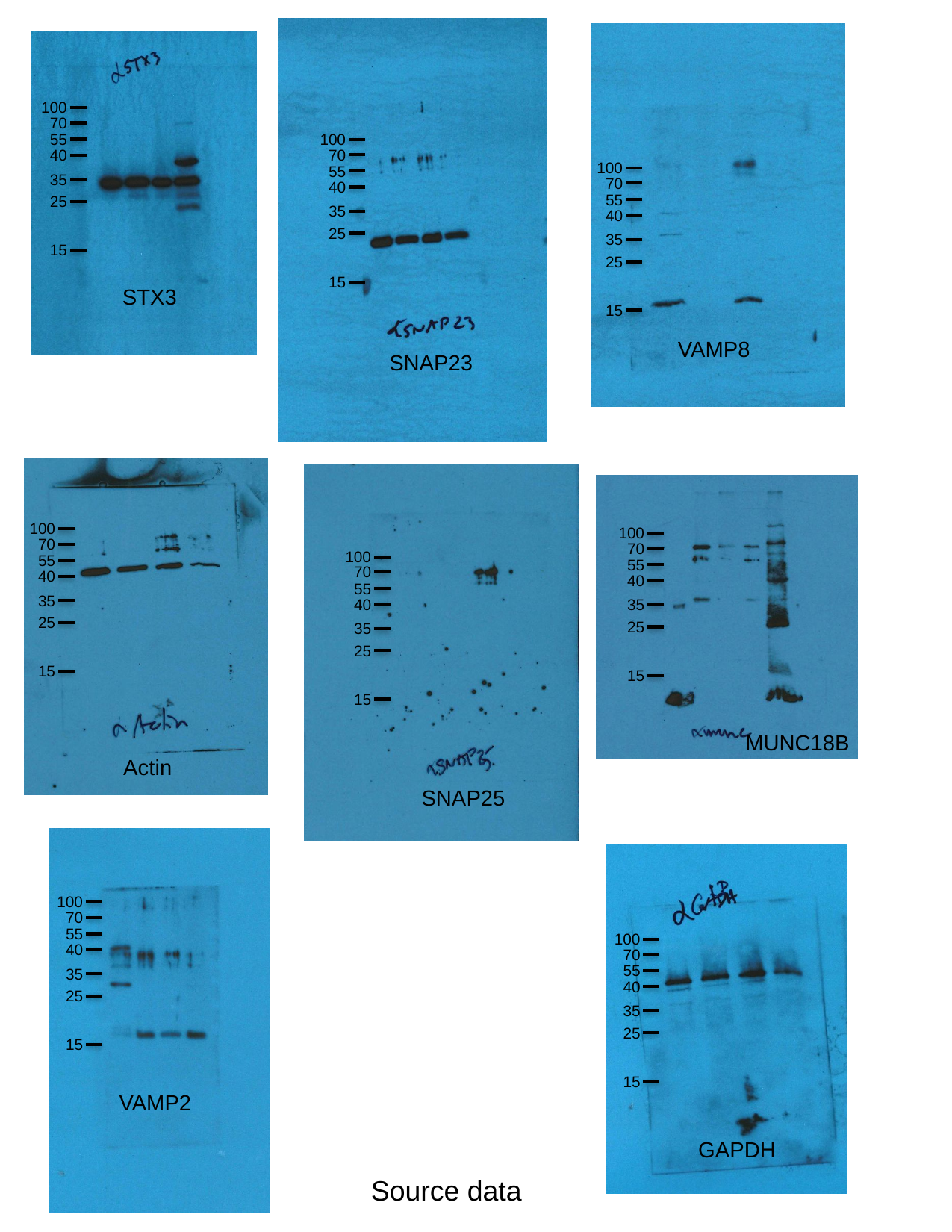

100
70
55
40
35
25
15
100
70
55
40
35
25
15
100
70
55
40
35
25
15
STX3
VAMP8
SNAP23
100
70
55
40
35
25
15
100
70
55
40
35
25
15
100
70
55
40
35
25
15
MUNC18B
Actin
SNAP25
100
70
55
40
35
25
15
100
70
55
40
35
25
15
VAMP2
GAPDH
Source data

Supplement: Supplementary file 2 — Source Data [file 41467_2019_11811_MOESM2_ESM.pptx]
